# Generation of mice with combined *Hexa* Gly269Ser KI or KO and *Neu3* KO alleles to create new models of GM2 gangliosidoses

**DOI:** 10.1242/bio.062045

**Published:** 2025-09-30

**Authors:** Emily N. Barker, Mehrafarin Ashiri, Jennifer T. Saville, Richard Hemming, Nikolas Furletti, Shreya H. Dhume, Shirley Yu, Elaine Anjos, Xiaoli Wu, Agnes Fresnoza, David C. Merz, Mike Jackson, Marc R. Del Bigio, Tabrez J. Siddiqui, Maria Fuller, Brian L. Mark, Barbara Triggs-Raine

**Affiliations:** ^1^ Department of Biochemistry and Medical Genetics, University of Manitoba, Winnipeg, Canada R3T 2N2; ^2^ Genetics and Molecular Pathology, SA Pathology at Women's and Children's Hospital, 72 King William Road, North Adelaide 5006, Australia; ^3^ School of Medicine, University of Adelaide, Adelaide 5005, Australia; ^4^ Physiology & Pathophysiology, University of Manitoba, Winnipeg, Canada R3T 2N2; ^5^ PrairieNeuro Research Centre, Kleysen Institute for Advanced Medicine, Health Sciences Centre, Winnipeg, Canada R3E 0Z3; ^6^ Pathology, University of Manitoba, Winnipeg, Canada R3T 2N2; ^7^ Children's Hospital Research Institute of Manitoba, 715 McDermot Ave, Winnipeg, Canada R3E 3P4; ^8^ School of Biological Sciences, University of Adelaide, Adelaide 5005, Australia; ^9^ Department of Microbiology, University of Manitoba, Winnipeg, Canada R3T 2N2

**Keywords:** Tay-Sachs disease, GM2 gangliosidosis, Mouse model, Adult onset, *Hexa*, *Neu3*

## Abstract

The GM2 gangliosidoses are lysosomal storage disorders exhibiting a spectrum of neurological phenotypes ranging from childhood death to debilitating adult-onset neurological impairment. To date, no mouse model harbouring a specific human mutation causing GM2 gangliosidosis has been created. We used CRISPR/Cas9 to generate knockin (KI) mice with the common adult-onset *Hexa* Gly269Ser variant as well as knockout (KO) mice with *Hexa* mutations expected to cause complete HexA deficiency. We also created *Neu3* KO alleles that combined with *Hexa* KO or KI alleles were expected to create acute and chronic models of GM2 gangliosidosis, respectively. However, both models accumulated GM2 ganglioside throughout the brain when compared to controls (CON), and exhibited progressive loss of reflexes, gait abnormalities, and premature death by 24 weeks of age. Although survival and behavioural phenotypes did not differ between KO and KI models, the KI model had substantial *Hexa* mRNA and evidence of GM2 turnover. This KI model will be useful for developing gene editing to correct the variant causing the Gly269Ser substitution and its novel biochemical phenotype suggests it may be suitable for testing therapies that treat partial β-hexosaminidase A deficiency.

## Teaser

**Summary:** Researchers generated and characterised mouse models of complete and partial HexA deficiency that are suitable for testing a broad range of therapies for Tay-Sachs disease.

## INTRODUCTION

The GM2 gangliosidoses are rare autosomal recessive disorders that result from the storage of GM2 ganglioside (GM2) primarily in the lysosomes of cells in the nervous system (Gravel et al., 2019). This accumulation is caused by mutations in either *HEXA* or *HEXB*, which encode the α- and β-subunits of β-hexosaminidase A (HexA) respectively; more rarely mutations are in the gene encoding the GM2 activator protein (*GM2AP*), which extracts GM2 from the lysosomal membrane. The active site of the HexA α-subunit then removes the terminal GalNAc and releases GM3 ganglioside.

HexA deficiency resulting from mutations in *HEXA* causes Tay-Sachs disease (TSD) and that from mutations in *HEXB* causes Sandhoff disease (SD). Disease severity is inversely correlated to the level of residual HexA activity, which determines the rate of GM2 accumulation (Leinekugel et al., 1992). It is estimated that 10% of normal HexA activity is needed to prevent the development of disease, whereas when there is no HexA activity acute-onset disease leads to death before 2-3 years of age. Only small increases in HexA activity (1-10% normal) lead to dramatic changes to phenotype, resulting in chronic forms of juvenile or adult-onset GM2 gangliosidosis that are less severe and exhibit a broad range of speech, motor and psychiatric problems (Toro et al., 2021).

The neurodegeneration observed in TSD and SD is due primarily to accumulation of GM2 ganglioside. Lyso-GM2, a de-acylated version of GM2, accumulates in patients with both TSD and SD and may also be pathogenic. Indeed, lyso-sphingolipids formed in Krabbe disease are more hydrophilic and cytotoxic than GM2 (Spassieva and Bieberich, 2016). Their increased hydrophilicity may facilitate access by HexA because patients with GM2AP deficiency, who have active HexA, accumulate GM2 but have normal levels of lyso-GM2, indicating that the enzyme was able to hydrolyze its terminal GalNAc (Kodama et al., 2011).

Many approaches have been explored to treat the GM2 gangliosidoses but there are currently no approved therapies (Toro et al., 2021). Several factors including HexA's instability, the need for both α- and β-subunits to generate HexA, and the requirement that it cross the blood brain barrier, have slowed progress toward treatment. However, recent advances are making enzyme replacement therapy and gene therapy for the GM2 gangliosidoses possible. An engineered HexA-like enzyme with ‘μ-subunits’ has been created, combining the active site of the *α*-subunit with the GM2AP binding regions and stability of the *β*-subunits (Tropak et al., 2016). The *μ*-subunit dimerizes, producing a stable homodimer named HexM, which has two active sites to break down GM2, and has been effective in treating mice with SD (Osmon et al., 2016). Recently, a modified form of HexM that includes an additional glycosylation site aimed at improving cellular uptake via mannose-6-phosphate has been developed (Lopez et al., 2025).

In mice, a *Hexb* knockout (KO) resulted in a model of SD that has been used extensively to study potential GM2 gangliosidoses therapies. These mice displayed neurological phenotypes including motor impairment, tremors, gait disturbances and seizures, as well as weight loss and increased brain weight compared to controls (Gulinello et al., 2008; Phaneuf et al., 1996; Sango et al., 1995). In contrast, mice with a KO of *Hexa* failed to produce a phenotype similar to human TSD (Miklyaeva et al., 2004) because of a ‘bypass’ pathway for GM2 degradation where sialic acid is removed from GM2 to create GA2 ganglioside, which then becomes a substrate for the β-subunit dimer HexB (Kolter and Sandhoff, 1998). Recently, an early-onset model similar to the TSD mouse was created by knocking out both *Neu3*, which encodes the neuraminidase that removes sialic acid from GM2, and *Hexa* genes (Demir et al., 2020; Seyrantepe et al., 2018). Knock-in (KI) mice harbouring specific human *Hexa* mutations that cause GM2 gangliosidosis have yet to be described.

Many missense mutations resulting in adult-onset GM2 gangliosidoses in humans cause alterations in protein folding that lead to their degradation. Development of mouse models with these specific missense mutations could be useful to test therapies for adult-onset forms of GM2 gangliosidoses. For example, pharmacological chaperone therapy uses small molecules to stabilize mutant proteins in the endoplasmic reticulum (ER), allowing them to be transported to the lysosome (Chiricozzi et al., 2014). Pyrimethamine (PYR), a competitive inhibitor of HexA, stabilizes monomers and assists in their trafficking through the ER, and has been shown to stabilize the *α*Gly269Ser-subunit, βArg505Gln-subunit and splice variant *α*IVS8-7G>A (Maegawa et al., 2007). Pharmacological chaperones can only be studied in models when some nascent protein is synthesized and begins folding in the ER; nonsense mediated decay of RNA transcripts could not be treated using this method. A model with residual protein could also provide a different background to study the immune response to novel engineered protein replacements or gene therapies that produce similar cross-reacting proteins.

In this study, we have used CRISPR/Cas9 to edit the genome of mice to generate the combined *Hexa*/*Neu3* double KO (dKO) that has been previously described as a model of TSD (Seyrantepe et al., 2018). Further, we describe a second model where the *Hexa* adult-onset mutation Gly269Ser (Navon et al., 1990) is combined with a *Neu3* KO (KIKO). Here, we characterise and compare the biochemical and neurological phenotypes of the two models.

## RESULTS

### Generation of *Hexa* KO, *Hexa* KI and *Neu3* KO alleles

We used CRISPR/Cas9 to generate KO and Gly269Ser KI alleles for *Hexa,* and KO alleles for *Neu3,* in C57BL/6N mice. The polymerase chain reaction (PCR)-based strategy described in the Materials and Methods, followed by Sanger sequencing of the targeted and surrounding regions, identified several founding mice transmitting alleles containing deletions in *Hexa* (Table 1) or *Neu3* (Table 2) or the *Hexa* c.805G>A (Gly269Ser) mutation (Table 1). Only those alleles harbouring out-of-frame mutations that were predicted to result in premature stop codons and therefore nonsense-mediated decay of the corresponding mRNA were considered suitable for generating the *Hexa* or *Neu3* KO mice. Each allele was given a unique designation using standard mouse nomenclature.

**Table 1.** Founding mice to generate *Hexa* KO and *Hexa* KI alleles

Mouse	*Hexa* mutation 1	Allele name	*Hexa* mutation 2	Allele name
1R	c.805G>A^1^	*Hexa^em2.1btr^*	ND	*Hexa^em1.1btr^*
1L	c.790Δttgtcct^2^	*Hexa^em1.2btr^*	c.790Δttgtcct^2^	*Hexa^em1.2btr^*
2L	c.805G>A^1^	*Hexa^em2.4btr^*	c.792ΔGT^2^	*Hexa^em1.3btr^*
2LL	c.805G>A^1^	*Hexa^em2.2btr^*	c.792ΔGT^2^	*Hexa^em1.3btr^*
5RR	c.794insT^2^	*Hexa^em1.5btr^*	c.794insT^2^	*Hexa^em1.5btr^*
2RRL	c.805G>A^1^	*Hexa^em2.3btr^*	ND	*Hexa^em1.4btr^*

1, knockin (KI) allele; 2, knockout (KO) allele; ND, not determined.

**Table 2.** Founding mice to generate Neu3 KO alleles

Mouse	*Neu3* mutation 1	Allele name	*Neu3* mutation 2	Allele name
1L	ND	*Neu3^em1btr^*	c.336ΔTGAGAGGTGC	*Neu3^em2btr^*
2R	c.334del18bp	*Neu3^em3btr^*	c.334del18bp	*Neu3^em4btr^*
3B	c.337insT	*Neu3^em5btr^*	c.336ΔTGinsA	*Neu3^em6btr^*
3RR	c.337insT	*Neu3^em7btr^*	c.326del17bp	*Neu3^em8btr^*
4L	c.330del18 bp	*Neu3^em9btr^*	c.333del11bp	*Neu3^em10btr^*

ND, not determined.

### Characterization of *Hexa* KO and *Hexa* KI mice

Founder mice were bred with wild type C57BL/6N mice to generate heterozygotes for each allele of interest. These included founders of *Hexa* alleles, *Hexa^em1.2^* (7 bp deletion) and *Hexa^em1.3^* (2 bp deletion) (Table 1), and two KI alleles for the c.805G>A mutation, *Hexa^em2.2^* and *Hexa^em2.3^* (Table 1). Intercrosses of heterozygotes for these alleles generated independent homozygous KO (*Hexa^em1.2/em1.2^*, *Hexa^em1.3/em1.3^*) and KI (*Hexa^em2.2/em2.2^*, *Hexa^em2.3/em2.3^*) lines. The presence of the expected mutations and absence of others in the targeted region were verified by Sanger sequencing when the alleles were in the homozygous state.

**Fig. 1.**
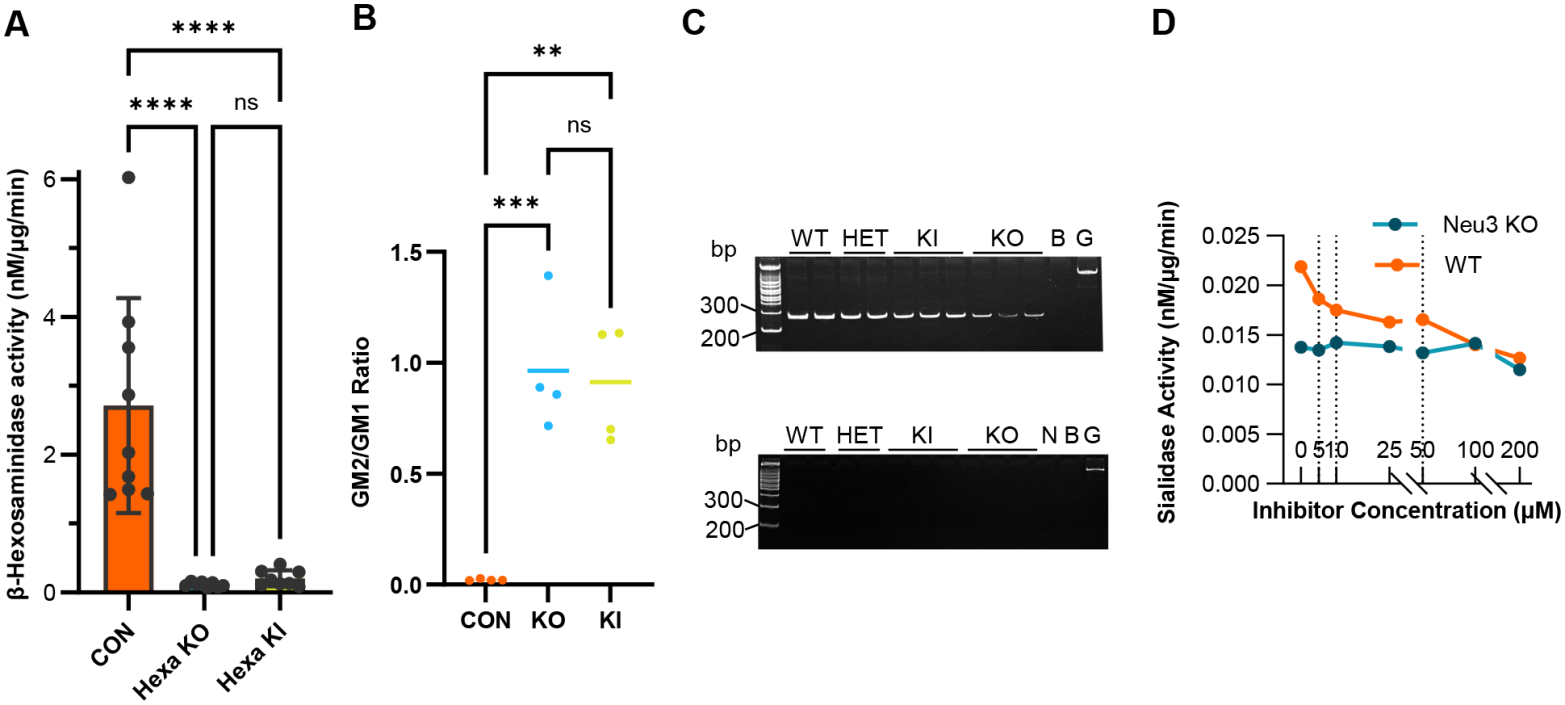
**Biochemical phenotypes of *HexA* KO, *Hexa* KI and *Neu3* KO mice.** (A) HexA enzyme activity. Brain lysates (∼20 µg protein) from CON (HET or WT, *n*=9, *Hexa* KO (*n*=8) or *Hexa* KI (*n*=8) mice were assayed using the synthetic substrate 4-MUGS. The average activity from three replicates was corrected for protein concentration in each sample. Columns represent the mean±s.d. for each group. In the brains of both models, HexA activity is significantly reduced (*P*<0.0001, ****) compared to CON. However, differences between models are non-significant (ns). Statistics were generated using a one-way ANOVA with Sidak's multiple comparison testing. (B) Semi-quantitative analysis of GM2/GM1 ratios in *Hexa* KO, KI and CON brains. Gangliosides isolated from mice brains (*n*=4 per genotype) as well as a ganglioside standard (GM1, GM2 and GM3 mix) were separated by TLC and visualized with orcinol. The GM2 and GM1 ganglioside levels in two or three independent experiments were analysed by densitometry and presented as a ratio of GM2 to GM1 ganglioside to correct for differences in the efficiency of the isolation. A one-way ANOVA followed with Tukey's multiple comparison testing found that the GM2/GM1 ratios did not differ between KO and KI groups, but differed significantly (*P*=0.0006 [KO], *P*=0.001 [KI]) from the CON group. (C) *Hexa* mRNA levels in the liver of *Hexa* KO and KI mice. Polyacrylamide gel electrophoresis of PCR products for *Hexa* cDNA and genomic DNA (G) (top panel) and CON without reverse transcriptase (bottom panel) that were generated using a forward primer in exon 7 and reverse primer in exon 8. The expected PCR product from the cDNA made from properly spliced mRNA transcripts is ∼300 bp, while genomic DNA with the intron is ∼1250 bp. The relative abundance of the PCR products was less for the *Hexa* KO (*Hexa^1.2^/Hexa^1.2^*; *n*=3) relative to *Hexa* KI (*Hexa^2.2^*/*Hexa^2.2^*; *n*=3), Het (*Hexa^1.2/wt^*; *n*=2) and WT (*Hexa^wt/wt^*; *n*=2) samples. CON for each sample without reverse transcriptase (RT) treatment (bottom) show that genomic DNA contamination was removed from the original RNA extracts. Column B shows a water blank for PCR and column N shows a water blank for RT reaction. Ladder: 100 bp ladder (Frogga Bio, DM001-R500). This figure is representative of one experiment. (D) Total sialidase activity in *Neu3* KO and WT mouse brain tissue. Sialidase activity was determined for 150, 225 and 300 µg protein, using 200 µM 4-MU-NANA substrate and different concentrations of the sialidase inhibitor zanamivir. Graphed values represent the mean sialidase activity in three technical replicates at each concentration corrected for µg protein in one experiment.

*Hexa* KO and KI mice were analysed for HexA activity using the synthetic α-subunit specific substrate 4-methylumbelliferyl β-N-acetylglucosamine 6-sulfate (4-MUGS). The average levels of HexA activity in *Hexa* KO and KI mouse brains did not differ from each other but were significantly decreased (*p*<0.0001) compared to brains from CON mice (Fig. 1A). CON mice included *Hexa* heterozygous (HET) and wild-type (WT) mice. To verify that the deficiency in HexA activity resulted in the accumulation of GM2 in the *Hexa* KO and KI brains, we used thin layer chromatography. Significant GM2 accumulation was found in both *Hexa* KO (*P<*0.001), and KI (*P*<0.01) brains compared to that in CON mice although KO and KI brains did not differ significantly (Fig. 1B). These data indicated that the KO and KI alleles both resulted in HexA deficiency and could not be differentiated based on residual HexA activity or accumulating GM2 levels. Further, a comparison of the residual HexA activity associated with the two independent KO (*Hexa^em1.2^* vs *Hexa^em1.3^*) and KI (*Hexa^em2.2^* vs *Hexa^em2.3^*) alleles showed no difference (Fig. S1) and therefore the *HexA^em1.2btr^* and *HexA^em2.2btr^* alleles were selected for use in subsequent studies.

Given the similar levels of HexA activity and GM2 accumulation in the *Hexa* KI and KO models, we decided to verify that significant levels of *Hexa* mRNA were detectable in the KI model. cDNA synthesized from equal quantities of total mouse liver RNA from *Hexa* KO, KI, and CON (HET and WT) mice were used to compare relative *Hexa* mRNA expression levels by reverse transcription/PCR. The KO mice had reduced levels of PCR product, indicating decreased *Hexa* mRNA compared to CON mice whereas the KI mice had levels of *Hexa* mRNA comparable to mice heterozygous for the KO allele, consistent with the presence of a normally synthesized *Hexa* transcript (Fig. 1C).

### Generation of *Neu3* KO mice

The *Neu3^em2^* (10 bp deletion) and *Neu3^em6^* (2 bp deletion/1 bp insertion) founders were chosen to generate *Neu3* null (KO) mice. Heterozygote intercrosses were performed to generate independent lines homozygous for two KO alleles (*Neu3^em2/em2^*, *Neu3^em6/em6^*). The presence of the expected mutations and the absence of other changes in the targeted region was verified by Sanger sequencing when they were in the homozygous state. To verify NEU3 deficiency, we measured total sialidase activity in a crude membrane fraction of *Neu3* KO brain using the synthetic substrate, 2′-(4-methylumbelliferyl)-α-D-N-acetylneuraminic acid (4-MU-NANA). Although there are four sialidases in mice, NEU3 has the lowest IC_50_ for the sialidase inhibitor Zanamivir and is the only family member localised to the membrane. In *Neu3* KO mice the membrane-associated sialidase activity without Zanamivir was lower than that of WT mice and did not decrease further with increasing concentrations of Zanamivir as was seen in the WT mice that have normal NEU3 activity (Fig. 1D).

### Generation of combined *Hexa* and *Neu3* deficiencies

Intercrossing mice with the *Hexa* KO or KI alleles and *Neu3* KO alleles was used to generate mice homozygous for *Hexa* KO and *Neu3* KO alleles (referred to as *HexaNeu3* double KO or dKO) as well as those homozygous for *Hexa* KI and *Neu3* KO alleles (referred to as *HexaNeu3* KIKO or KIKO). The impact of the two different *Neu3* alleles (*Neu3^em2^* and *Neu3^em6^*) on survival of dKO, KIKO, and CON mice was compared (Fig. S2A and B). No difference in the survival was observed and therefore we used mice with either *Neu3* allele for the comparison of dKO and KIKO models. dKO and KIKO mice of both sexes could produce litters if the other parent was heterozygous for *Hexa.* We did not test homozygous intercrosses.

### Survival of *HexaNeu3* dKO and *HexaNeu3* KIKO mice

**Fig. 2.**
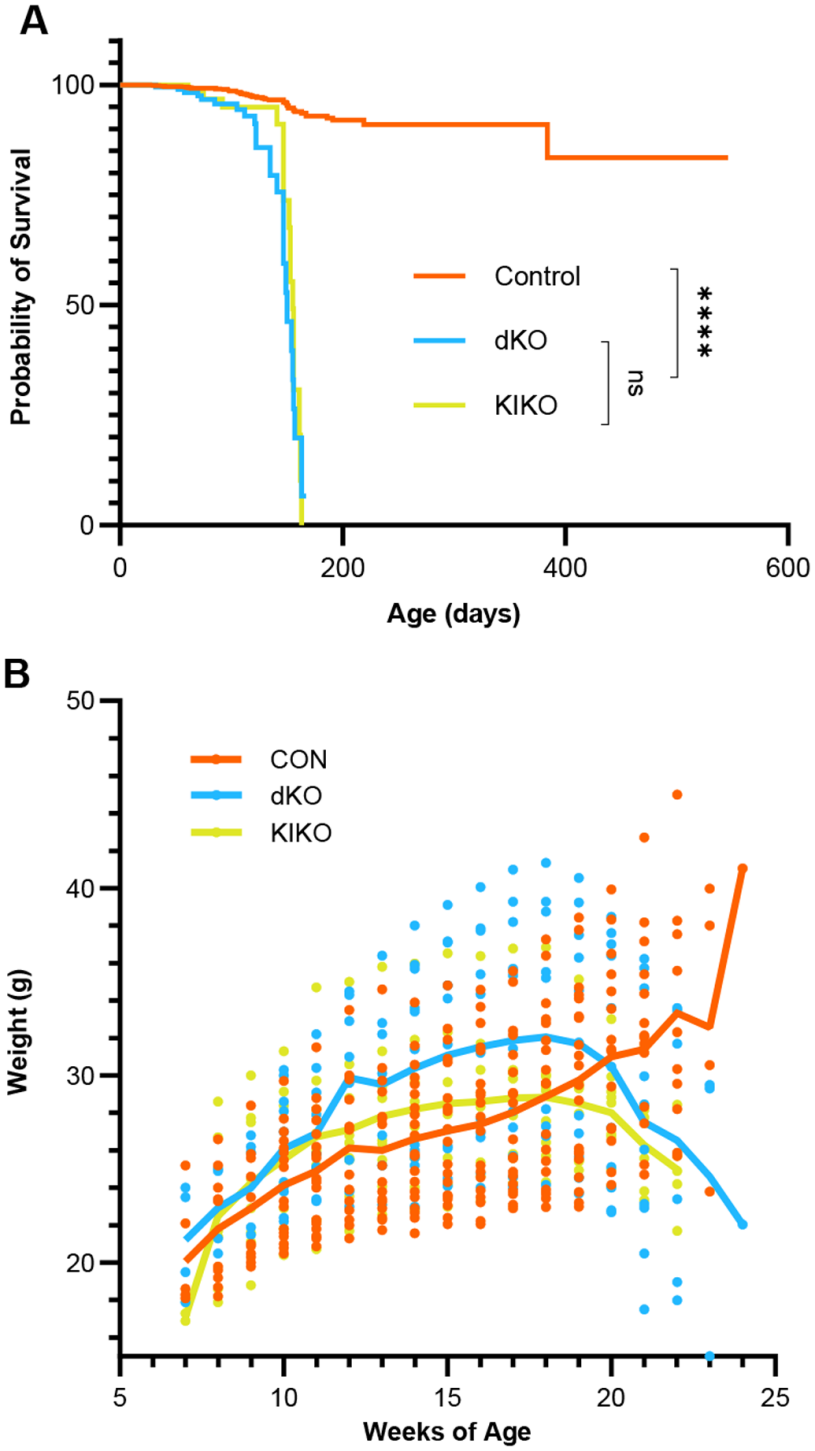
**Weight and survival analysis of *HexaNeu3* dKO and *HexaNeu3* KIKO mice.** (A) Kaplan–Meier survival analysis. Mice were monitored from 7-10 weeks after birth for HEP. The difference in survival probability between dKO or KIKO compared to CON (*n*=68 for all groups) was statistically significant *P*<0.0001 (****), however there was no significant difference (ns) between dKO and KIKO groups. Statistics were conducted using log-rank tests for individual group comparsions and *P*-values adjusted for multiple comparisons using Holm–Sidak method (α=0.05). (B) Weight trajectory of dKO, KIKO and CON mice. The weight of mice was measured weekly starting between 7 and 10 weeks of age until the HEP was reached. Lines connect the average weight for dKO (blue, *n*=6-11), KIKO (green, *n*=5-9), and CON (orange, *n*=11-20). The number of mice at some time points differ because some of the mice were not entered into the study until 10 weeks or were euthanised early.

Kaplan–Meier survival analysis showed a significant difference (*P<0.0001*) in the percent survival of dKO or KIKO mice compared to healthy CON but no significant difference between the dKO and KIKI mice (Fig. 2A), which had a median survival of 150 and 155 days respectively. The humane endpoint (HEP) was most often reached due to weight loss. Weight was monitored weekly starting between 7 and 10 weeks and more frequently when the HEP was near. Both dKO and KIKO mice gradually gained weight to surpass CON mice, peaking at 18 weeks with a weight of 32.1±6.3 g and 28.9±3.6 g respectively (Fig. 2B). Weight declined thereafter at ∼1 g/week until HEP with similar survival and weight gain/loss for both sexes (Fig. S3A and B). Occasionally animals were euthanised due to injury from fighting or self-mutilation. A subset of young mice in both the dKO (4.9% of total) and KIKO (5.1% of total) colonies died between 7-29 days, as described previously for *HexaNeu3* dKO mice (Seyrantepe et al., 2018), and were excluded from the survival analysis.

### Assessment of neurological phenotypes of *HexaNeu3* dKO and *HexaNeu3* KIKO mice

**Fig. 3.**
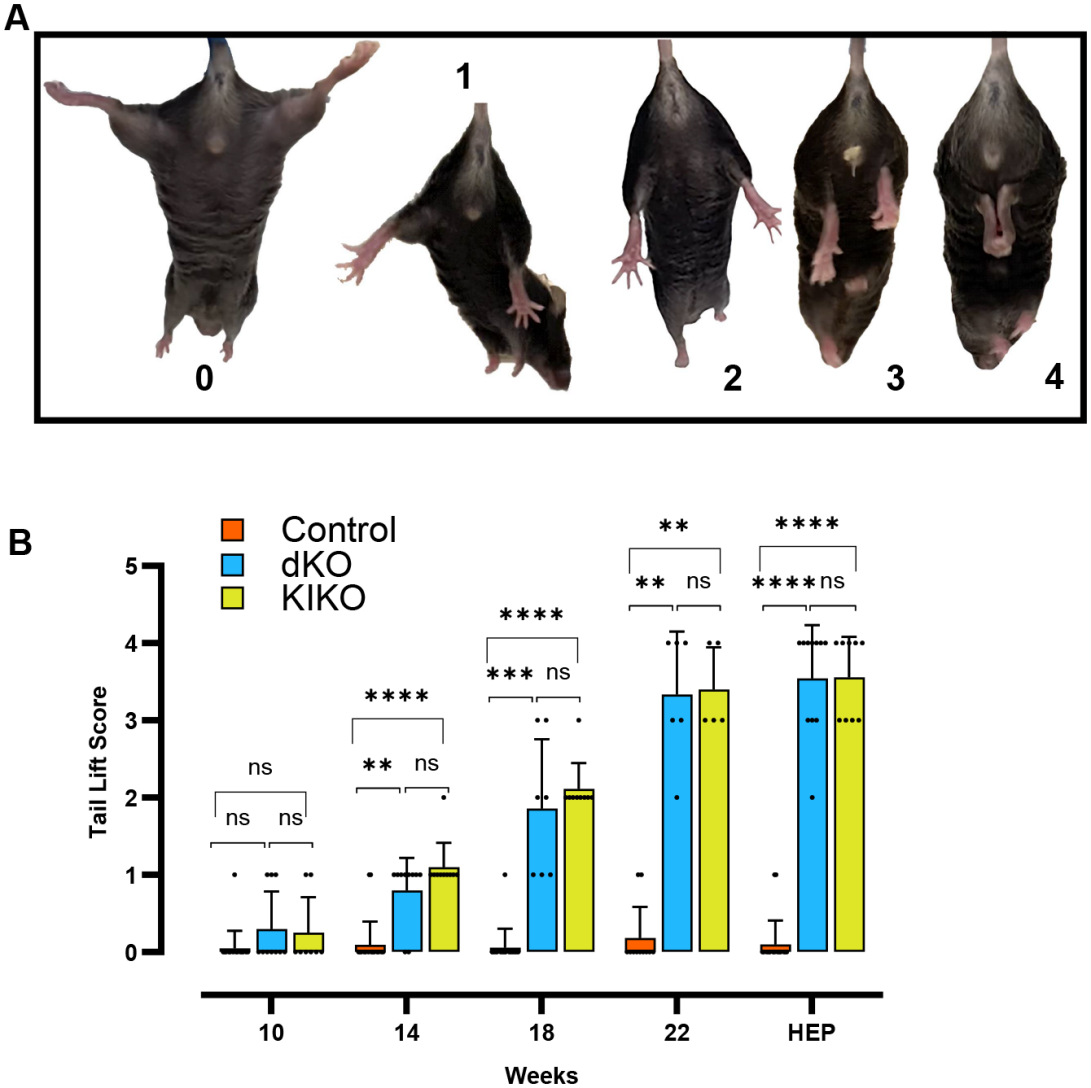
**Analysis of tail lift (TL) scores in dKO, KIKO and CON mice.** (A) Representative images of hindlimb flexion with corresponding TL score. A written description of the scoring is provided in Table S1. (B) TL score at 10, 14, 18, 22 weeks and HEP. TL scores were assigned during a 5 s tail suspension according to Table S1. Kruskal–Wallis test with Dunn's multiple comparisons was conducted for mean±s.d. TL test scores of dKO (blue, *n=*6-11), KIKO (green, *n=*5-9), and CON (orange, *n=*11-20) at 10, 14, 18, 22 weeks of age and at HEP (19-24 weeks of age). dKO and KIKO mice did not display significant hindlimb flexion (average score between 0 and 1) at 10 weeks compared to CON. By 14 weeks both dKO and KIKO scores increased significantly compared to CON without immobility or limb clasping. Comparisons at 22 weeks and HEP show all dKO and KIKO mice displayed median scores above 2 with abnormal hindlimb flexion. Comparisons of all animals at HEP indicated the age- and sex-matched CON mice continued to have a normal extension response and scores less than or equal to 1. *(*P*<0.05), **(*P*<0.01), ***(*P*<0.001), ****(*P*<0.0001), ns (nonsignificant).

Neurological assessment included scoring of hindlimb extension reflex, open field test (OFT) and gait analysis. The hindlimb extension reflex was assessed weekly between 10 and 22 weeks of age using a tail lift test and scored between 0 and 4 using a reference grid (Table S1) and visual examples (Fig. 3A). Both dKO and KIKO mice displayed a reduction or loss of reflex that became progressively worse until the HEP. Repeated scoring from 10-22 weeks revealed a progressive and significant reduction in extension reflex beginning at 14 weeks and eventual flexion response by HEP in dKO (*P<*0.0001) and KIKO (*P<*0.0001) animals (Fig. 3B). CON mice maintained normal hindlimb extension reflex, although occasionally over conditioned mice had scores of 1.

**Fig. 4.**
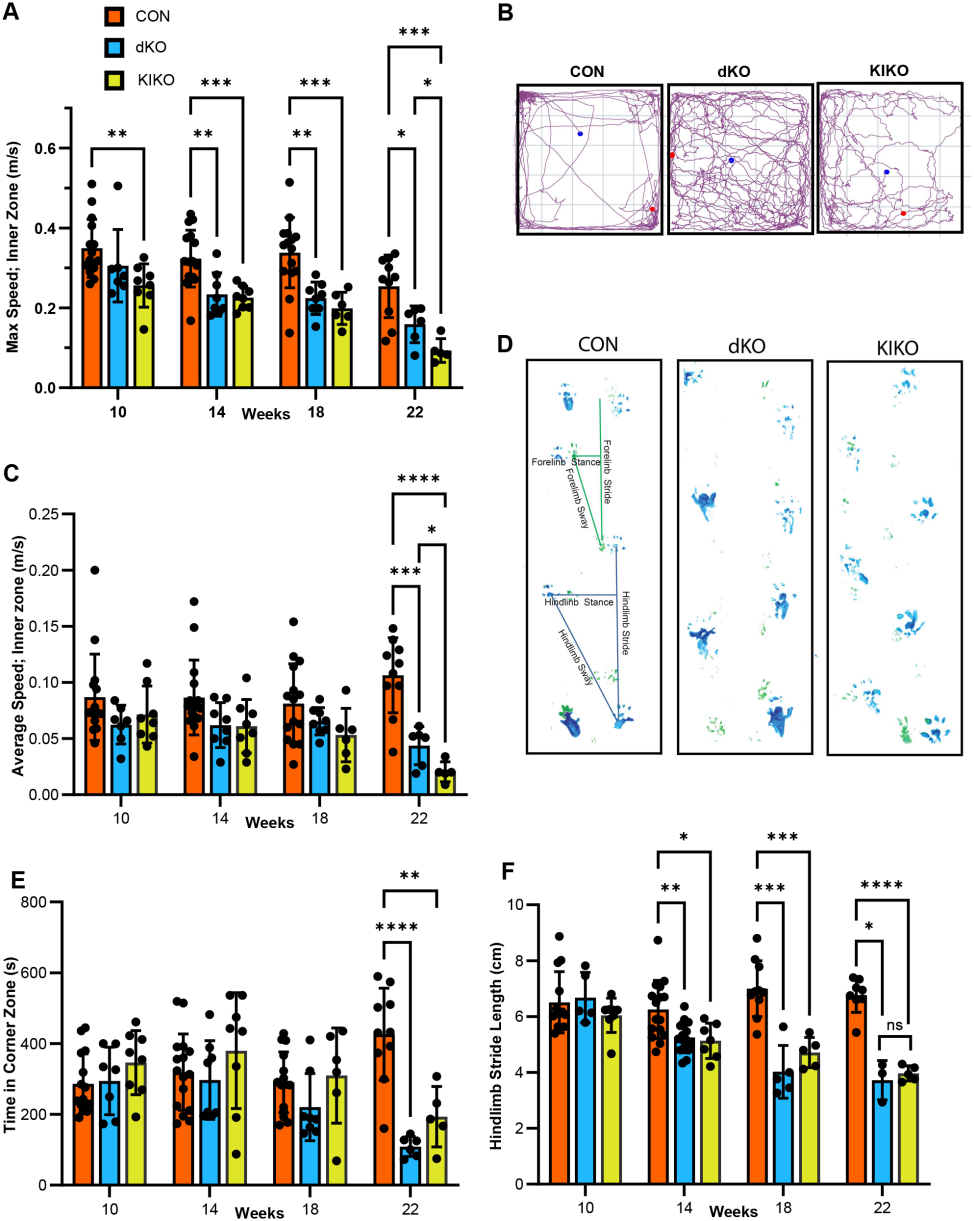
**Assessment of neurological phenotypes in dKO, KIKO and CON mice.** (A) Maximum speed (m/s) at 10, 14, 18 and 22 weeks. The maximum speed was tested in the OFT when mice were in the inner zone (50×50 area, 10 cm from each wall of arena). Each column represents the mean±s.d. maximum speed. The decrease in maximum speed of both dKO (*n*=6 to 8) and KIKO (*n*=5 to 8) mice was significant when compared to CON (*n*=10 to 16) mice at 10 weeks and both dKO and KIKO mice were significantly impaired compared to CON at 22 weeks. Statistics were conducted with a mixed-effects model (REML) using Geisser–Greenhouse correction, with Tukey's multiple comparisons test for each pairing) *(*P*<0.05), **(*P*<0.01), ***(*P*<0.001), ****(*P*<0.0001), ns (nonsignificant). (B) Track plots at 22 weeks. Track plots were generated using ANYmaze video tracking software. The beginning and end of the track is indicated by the blue and red circles respectively. Increased use of the inner zone, along with gait and balance disturbances were revealed in the dKO and KIKO mice. (C) Average speed (m/s) at 10, 14, 18 and 22 weeks. As described in A, the average speed was tested in the inner zone of the area. Each column represents the mean±s.d. Statistics were as described for A. (D) Representative gait prints at 22 weeks. Gait prints were used to measure stride length, stance and width, as illustrated in the example. Gait became disorganised by 22 weeks in both dKO and KIKO models compared to CON mice with reduction in stride length and stance width measurements. (E) Time in the corner zone (s) at 10, 14, 18 and 22 weeks. The time spent in the corner zone (10×10 area, in each corner) of the open-field maze was measured in seconds during the 600 s test. Each column represents the mean±s.d. of total time spent by mice in each group at each age. The decrease in time spent in the corners of both dKO (*n*=6 to 8) and KIKO (*n*=5 to 8) mice was significant when compared to CON (*n*=10 to 16) mice at 22 weeks. Statistics were conducted with a mixed-effects model (REML) using Geisser–Greenhouse correction, with Tukey's multiple comparisons test for each pairing *(*P*<0.05), **(*P*<0.01), ***(*P*<0.001), ****(*P*<0.0001), ns (nonsignificant). (F) Hindlimb stride length at 10, 14, 18 and 22 weeks. The stride length was determined from gait prints as shown in D. Three measurements of hindlimb stride were done for each gait print and the mean was used for analysis. Each column represents the mean±s.d. hindlimb stride length at that point in time. Both dKO (*n*=3 to 16) and KIKO (*n*=5 to 8) mice have significantly shortened hindlimb stride length (cm) compared to CON (*n*=8 to 16) mice from 14-22 weeks. Statistics were conducted using a mixed-effects model (REML) using Geisser–Greenhouse correction, with Tukey's multiple comparisons test for each comparison.

The open field test (OFT) was used to examine mobility and anxiety-related behaviours. Mobility measures were taken of maximum and average speed in the inner zone (center 40 cm^2^), as well as distance travelled over the duration of the test. Maximum speed was the most impacted measure of mobility, with significant differences between CON and KIKO mice even at 10 weeks (Fig. 4A, *P<*0.01). Significant deterioration to maximum speed in both models was observed at 14 weeks (dKO; *P*<0.01, KIKO; *P*<0.001) and this trend continued until 22 weeks (Fig. 4A). Distance travelled between CON, dKO and KIKO mice showed no statistically significant difference except between the dKO and KIKO mice at 22 weeks (Fig. S4A), despite other obvious changes to mobility in both models (Fig. 4B). The significant differences detected between models at 22 weeks may be due to the small number of animals in these groups by this time point. Given that groups did not differ in their distance travelled (Fig. S4A) but did differ in their maximum speed (Fig. 4A), this suggested that the dKO and KIKO models are consistently active albeit at a reduced speed. Indeed, the average speed of the dKO and KIKO mice in the inner zone did not differ significantly from that of CON mice until 22 weeks of age (Fig. 4C). Additionally, time spent while mobile in the maze was not significantly different among groups except for between CON and KIKO at 14 weeks (*P*<0.05) (Fig. S4B).

Changes to anxiety-related or exploratory behaviour were determined by time spent in various zones. Mice that spend more time in the inner zone have decreased anxiety-like behaviour or increased exploratory behaviour compared to those that spend time in the outer zone or corners (Seibenhener and Wooten, 2015). The inner zone was defined as the center 40 cm^2^, outer zone as the 10 cm perimeter of the open field, and the corners as a 10 cm^2^ area in each of the four corners. A decrease in thigmotaxis, or tendency to travel along walls in the outer zone, was observed in dKO and KIKO mice at around 14 weeks and continued to gradually diminish until 22 weeks. Indeed, the track plots of the mice obtained during the OFT clearly show dKO and KIKO mice spend more time occupying the inner zone than CON mice (Fig. 4B). CON animals also spent an increased amount of time in corners compared to dKO (*P*<0.0001) and KIKO (*P*<0.01) animals at 22 weeks (Fig. 4E).

Balance and gait difficulties among dKO and KIKO mice were also observed. Track plots taken during the OFT show a distinct wave pattern in dKO or KIKO mouse paths at 22 weeks compared to CON (Fig. 4B), likely due to the gait difficulties. dKO and KIKO animals appeared to have normal gait patterns for all measures compared to CON at 10 weeks, before patterns became increasingly disorganized by 22 weeks (Fig. 4D). This change in gait included a decrease in stride length (Fig. 4F), which was significant for dKO and KIKO mice at 14 weeks (*P*<0.01, and *P*<0.05 respectively). Stride lengths continued to shorten over time, and by 22 weeks average hindlimb stride length had decreased by 45% and 41% for dKO and KIKO mice respectively when compared to CON (dKO, *P<*0.05; KIKO, *P<*0.0001). Hunched posture with elevated tail while walking and tremors were a common observation in both dKO and KIKO mice. These observations were difficult to quantify but were consistent across various observers and animals.

### Ganglioside levels in brains of *HexaNeu3* dKO and *HexaNeu3* KIKO mice

**Fig. 5.**
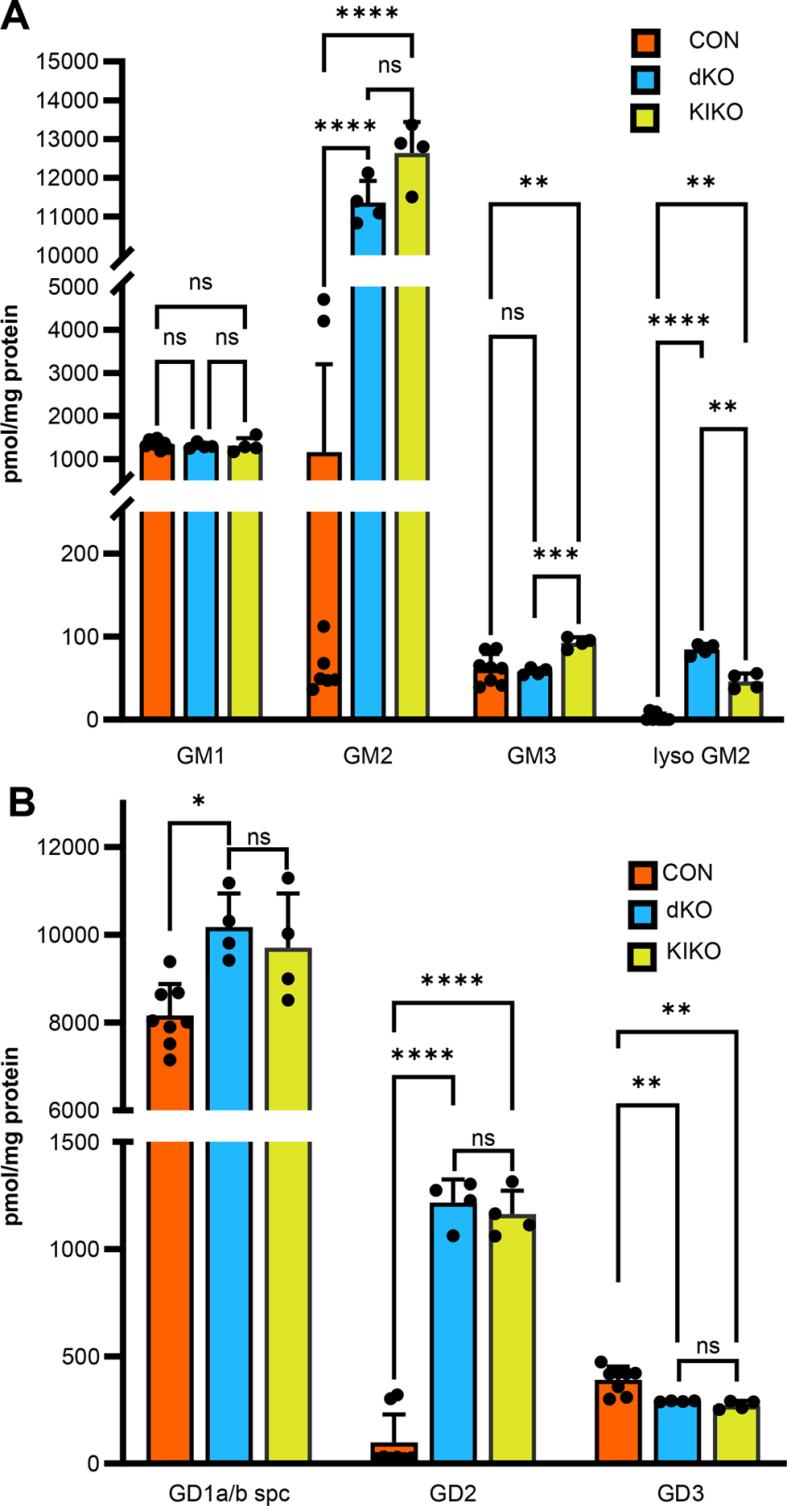
**LC-ESI-MS/MS analysis of brain gangliosides from dKO, KIKO and CON mice.** (A) Total quantification of monosialo ganglioside series. Total of isoforms with fatty acid lengths for each class of the monosialo ganglioside series in expressed as mean±s.d. pmol/mg protein from the brains of dKO (*n*=4), KIKO (*n*=4) and CON (*n*=5) mice. dKO and KIKO showed significant GM2 (*P*<0.0001) and Lyso-GM2 accumulation (dKO [*P*<0.001]; KIKO [*P*<0.01]) when compared to CON mice. KIKO mice showed a significant increase in GM3 (*P*<0.001) and a decrease in Lyso-GM2 (*P*<0.01) compared to dKOs. (B) Total quantification of disialo ganglioside series. The total of isoforms with differing fatty acid lengths for each class of disialo ganglioside is expressed as mean±s.d. pmol/mg protein from the brains of dKO, KIKO and CON mice. Both dKO and KIKO mice had significantly increased GD2 (*P*<0.0001) and decreased GD3 (*P*<0.01) compared to CON mice. Additionally, dKO mice had significantly increased GD1a/b series gangliosides when compared to CON (*P*<0.05). Statistics for both A and B were conducted using a two-way ANOVA with the Geisser–Greenhouse correction, with Tukey's multiple comparisons test for each comparison *(*P*<0.05), **(*P*<0.01), ***(*P*<0.001), ****(*P*<0.0001), ns (nonsignificant).

dKO and KIKO mouse brains were consistently a pale-yellow colour compared to CON brains as has been described previously for mice with GM2 accumulation (Seyrantepe et al., 2018). Mass spectroscopy of gangliosides isolated from the brains of dKO, KIKO and CON mice was used to quantify the monsialo- and disialo ganglioside content (Table S2). CON mice were HET at either the *Hexa* or *Neu3* locus and included mice of different genotypes (*Hexa^em1.2/+^Neu3^em2/em2^* and *Hexa^em2.2/+^Neu3^em6/em6^* [*Hexa^+/−^Neu3^−/−^*; *n*=5], *Hexa^em2.2/em2.2^Neu3^em6/+^* [*Hexa^−/−^Neu3^+/−^*; *n*=2], and *Hexa^em2.2/+^Neu3^em6/+^* [*Hexa^+/−^Neu3^+/−^*; *n*=1]). Data were analysed with CON values grouped for simplicity (Fig. 5A and B) as well as separately by genotype (Fig. S5A and B). The GM2 levels in the dKO and KIKO mice were elevated 11-13-fold (*P*<0.0001) when the CON samples were grouped (Fig. 5A). However, if the dKO and KIKO GM2 values were compared only to those of *Hexa^+/−^Neu3^−/−^* CON mice, the increase was about ∼200 fold (Fig. S5A, dKO, *P*<0.0001; KIKO, *P*<0.001). This difference is because of the accumulation of GM2 in one genotype of CON mice; *Hexa^−/−^Neu3^−/−^* mice accumulated ∼70-fold GM2 compared to *Hexa^−/−^Neu3^+/−^* (*P*<0.001) (Fig. S5A). Interestingly, there was no significant difference between the dKO and KIKO GM2 levels (Fig. 5).

The lyso-GM2 levels were also significantly increased in both dKO (*P*<0.0001) and KIKO (*P*<0.01) models compared to that in CON mice, but the levels in dKO mice were significantly higher than that of KIKO mice (*P*<0.01), suggesting the presence of a low level of HexA activity and GM2 breakdown in the KIKO model (Fig. 5A). Consistent with this, the levels of GM3 ganglioside were significantly increased in the KIKO compared to dKO (*P*<0.001) and CON (*P*<0.01) mouse brains (Fig. 5A). GM1 levels in dKO and KIKO mice did not differ from the CON (Fig. 5A).

The results of the disialo-ganglioside analysis are shown in Fig. 5B and Fig. S5B. GD1a/b gangliosides that are derived from GM1 were significantly increased in dKO mice compared to CON (*P*<0.01); KIKO mice also displayed increased GD1a/b ganglioside, but it did not reach significance. GD2, which is a product of GM2, was significantly increased in both models compared to CON but did not differ between models (Fig. 5B), whereas GD3, was significantly decreased in dKO and KIKO mice compared to CON. There were also differences in the gangliosides that accumulated in the CON mice that had differences in their allele combinations (Fig. S5A and B). Mice that were *Hexa* KO and *Neu3* HET, i.e. had one *Neu3* WT allele (*n*=2) exhibited significant differences despite the small sample size. These included increased GM2, decreased GM3, increased GD2 and decreased GD3 compared to CON with one *Hexa* WT allele (*n*=4) (Fig. S5A and B). Among the gangliosides, the most abundant fatty acid chain lengths were 36:1, followed by 38:1.

### Distribution of GM2 ganglioside in the brains of dKO and KIKO mice

To examine the distribution of GM2 ganglioside in mouse brain regions, matched slides for dKO (*n*=3) or KIKO (*n*=3) and CON (*n*=6) were selected based on anatomical features including cerebellar nuclei, hippocampal formation and ventricle position using the Allen Mouse Brain Atlas145 (mouse.brain-map.org). Brains were immunostained using a monoclonal antibody against GM2 (Miyake et al., 1988) and counterstained with methyl green.

GM2 ganglioside was detected in the brains of all mice but the number and intensity of positively staining cells, most obviously in cytoplasmic organelles of large neurons, was higher in most parts of the brains of dKO and KIKO mice compared to CON (Fig. S6A and B). Consistent with the previous studies of *Hexa^−/−^Neu3^−/−^* mice (Seyrantepe et al., 2018), GM2 accumulation was evident in cells of the cerebellum of the dKO (Fig. S6A,B and F) and KIKO (Fig. S6A,D and H) as well as the hippocampus of the dKO (Fig. S6A,J) and KIKO (Fig. S6A,L). The levels of staining for GM2 in cells of the cerebral cortex was more diffuse (Fig. S6B,F and H) than that in several other regions of the brain. For example, significant GM2 accumulation was also found in the thalamus (Fig. S6A,N and P), medulla (Fig. S6A,R and T), pons (Fig. S6B,B and D), hypothalamus (Fig. S6B,J and L) and midbrain (Fig. S6B,N and P).

**Fig. 6.**
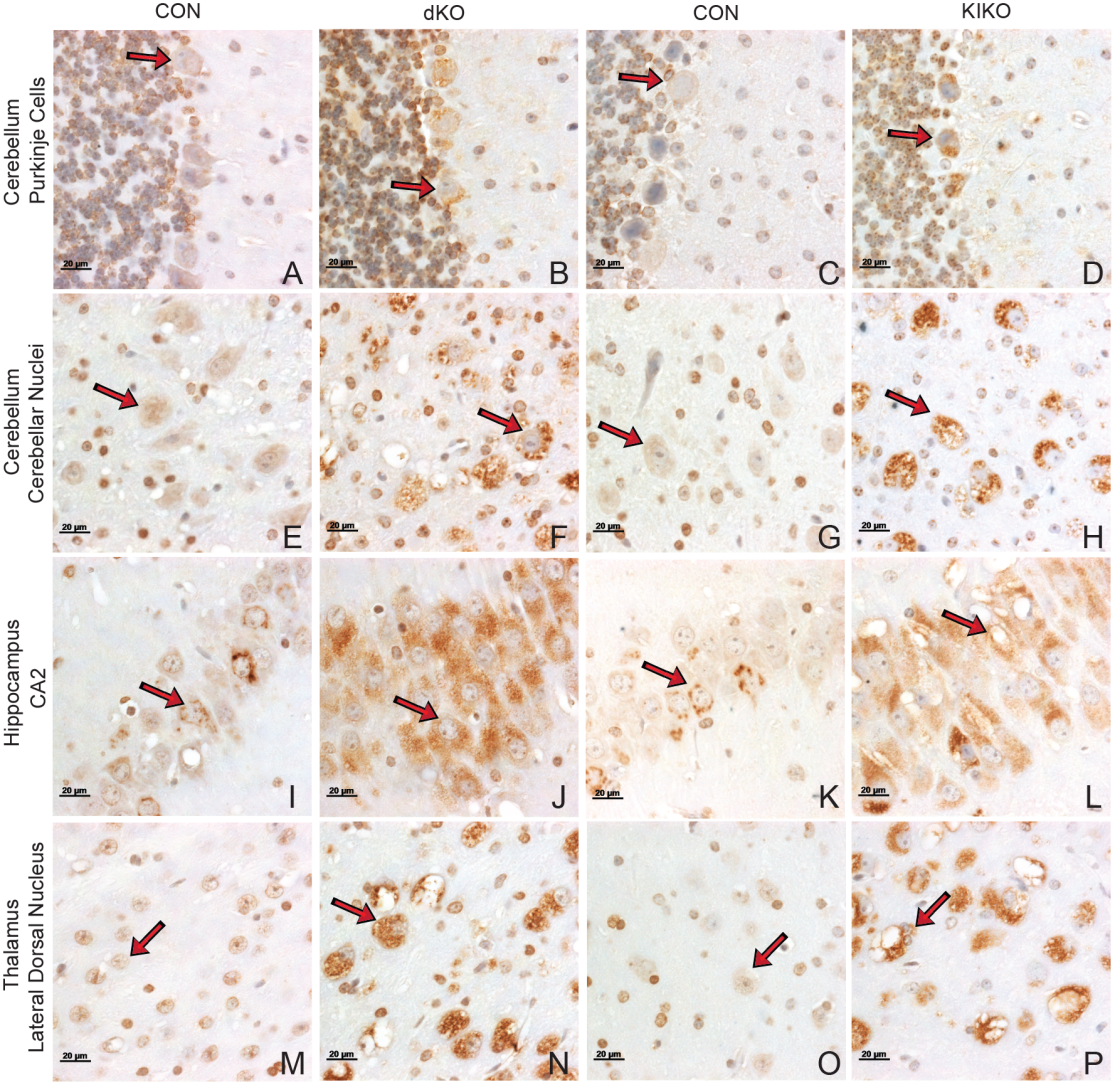
**Immunohistochemical detection of GM2 ganglioside in dKO, KIKO and CON brains.** Sections were selected from similar areas of the brain for matched pairs. Sections for each matched pair were stained together and photographed at 63× using a Zeiss Axioimager equipped with ZEN software. Many images are taken from within the same field of view as the 20× images shown in Fig. S6A. Specifically, data in Fig. 6, panels E,F,G,I,J,N, and O are also shown in Fig. S6A, panels A,B,C,I,J,N, and O respectively. The presence of a red-brown staining signal for GM2 ganglioside was present in CON brains but much more broadly and abundantly present in the sections from the dKO and KIKO brains. Arrows indicate cells that have low levels of GM2 in CON (A,C,E,F,I,K,M,O), compared to the higher levels of GM2 present in vacuolated cells (B,D,F,H,J,L,N,P). Arrows in panels A-D identify Purkinje cells. Scale bars: 20 µM. These images are representative of those from three different pairs of animals.

GM2 accumulation that was observed in dKO and KIKO mice caused engorgement of neurons comparable to that seen in other animal models and patients with GM2 gangliosidoses (Fig. 6; Fig. S6C). Higher magnification of the cells showed GM2 accumulation in the Purkinje cells of the cerebellum (Fig. 6B) as well as a reduced number of Purkinje cells compared to CON (Fig. 6A and C). This loss of Purkinje cells in GM2 gangliosidosis models has previously been described (Demir et al., 2020). Distinctive granular accumulation, likely within lysosomes, was evident in neurons of the cerebellum (Fig. 6F and H) and thalamus (Fig. 6N and P). The GM2 accumulation in neurons of the hippocampus CA2 region was more diffuse (Fig. 6J and L). Significant GM2 accumulation was also observed in the cell bodies of neurons in the medulla (Fig. S6C,B and D) and to a lesser extent in neurons of the inferior colliculus (Fig. S6C,F and H). Sections which were not incubated with the primary antibody were processed as a control and showed negligible brown staining (Fig. S6C,I-L).

## DISCUSSION

We have generated a novel KIKO mouse model of GM2 gangliosidosis with a KI of the well-known adult-onset *HEXA* mutation (Gly269Ser) and a KO of the sialidase encoding gene, *Neu3.* Mice with a KO of both the *Hexa* and *Neu3* genes (dKO), that were previously described to have a severe TSD-like phenotype (Demir et al., 2020; Seyrantepe et al., 2018), were also generated during this study by combining the *Neu3* KO allele with a newly generated *Hexa* KO allele. Although the KI mutation we introduced did not produce enough residual enzyme activity to extend survival or create a chronic model of GM2 gangliosidosis (Fig. 2A), it did introduce novel biochemical changes that indicated some residual HexA activity was present in mice with the Gly269Ser-encoding *Hexa* allele (Figs 1C and 5A,B).

Our studies showed that both the dKO and KIKO models had an average survival of 22 weeks (Fig. 2A). Previously, the *Hexa^−/−^Neu3^−/−^* model was typically found to survive for only 3-4 months, with occasional survival to 5 months (Seyrantepe et al., 2018). This may be explained by background differences in the mice or possibly the additional accommodations that we made to facilitate access to food and water when mobility was decreased. The longer survival time that we observed makes these models slightly less severe than the *Hexb^−/−^* models of SD that were found to have a HEP of 18-20 weeks using similar HEP criteria (Sango et al., 1995; Walia et al., 2015). Most dKO and KIKO mice in our study were euthanised at HEP due to weight loss, even though food and water were readily available and general mobility was preserved. KIKO mice began to lose weight after 18 weeks compared to 16 weeks for dKO (Fig. 2B), suggesting that KIKO mice may have a later onset of this dysfunction, however this did not extend survival. Interestingly, dKO and KIKO individuals of either sex could produce offspring, despite previous reports of dKO mice being sterile (Seyrantepe et al., 2018).

The KIKO and dKO models that we generated recapitulated GM2 gangliosidosis progression in humans with initial normal development compared to littermates, followed by progressive neurodegeneration (Figs 3 and 4), mobility deterioration (Fig. 4), reappearance of reflexes from previous stages of development (Fig. 3) and behavioural changes (Fig. 4). Accumulation of GM2 in the central nervous system was extensive, but particularly profound in the cerebellum and thalamus, areas involved in motor coordination and sensory signal relay (Heck et al., 2023). This accumulation was the probable cause of disease progression and was seen with similar severity in both dKO and KIKO models (Figs 5 and 6, Figs S5 and S6). These findings were also consistent with previous extensive descriptions of the dKO model (Can et al., 2022; Demir and Seyrantepe, 2024; Demir et al., 2020; Seyrantepe et al., 2018).

One of the earliest signs of neurological deterioration in our models was the loss of the hindlimb extension reflex. Loss of reflexes or regression to a previous developmental stage is a sign of a severe neurological disorder, and in GM2 gangliosidoses across multiple onsets, exaggerated reflexes and reappearance of Babinski reflex is common (Jamrozik et al., 2013; Toro et al., 2021). In mice, the hindlimb extension reflex can be initiated by visual stimuli and tactile sensations in the vibrissae or paws. Limb clasping in 2-week-old mice is slowly converted to extension once the eyes are open, although tactile stimuli to the vibrissae is sufficient to cause hindlimb extension throughout the postnatal period (Lalonde and Strazielle, 2011). In dKO or KIKO mice, normal extension reflex was observed until about 14 weeks, before progressively becoming weaker and finally regressing to limb clasping in both models. Loss of the hindlimb extension reflex has been associated with models where cerebellar degeneration, denervation or dysfunction are prominent features (Lalonde and Strazielle, 2011). In both our dKO and KIKO models, one of the most prominent areas of GM2 accumulation in the brain was the cerebellar nuclei, suggesting damage to this area contributed to the abnormal reflexes. Additionally, our models exhibited abundant GM2 accumulation in the large neurons in the pons and medulla that are responsible for relaying somatic sensory information from the hindlimbs to the brain (Treuting et al., 2017).

Locomotor activity and behavioural changes were analysed in both dKO and KIKO models using the open field test (Fig. 4; Fig. S4). Consistent with previous findings for the dKO model (Demir et al., 2020), locomotor activity decreased in both models. However, in contrast to their findings, we observed a decrease in thigmotaxis suggesting lowered anxiety or increased exploratory behaviour (Seibenhener and Wooten, 2015). Thigmotaxis is reliant on tactile sensations such as whisker contact with the walls of the maze, along with visual stimuli (Kulesskaya and Voikar, 2014; Prut and Belzung, 2003). Damage to these, or the communication between the sensory neurons and the higher processing centres, can result in decreased thigmotaxis. The previously reported dKO animals did exhibit GM2 accumulation in the retina (Bardak et al., 2015; Demir et al., 2020), suggesting visual problems, though we did not investigate this aspect of disease. Thus, while decreased thigmotaxis may indicate decreased anxiety, other factors including loss of visual and tactile sensations may explain these decreases. The basis for the opposing findings is unclear but could have been influenced by the 10 min (versus 5 min) test time used in our study.

GM2 ganglioside accumulation in both dKO and KIKO models was clearly demonstrated by both immunohistochemistry and mass spectrometry (Fig. 5, Fig. S5, Fig. 6 and Fig. S6). Like the histological findings in SD mice, GM2 was detected throughout the brain, including in the cerebellar Purkinje and granular cells, which are spared in *Hexa^−/−^* mice (Sango et al., 1995). Although the KIKO model appeared to have reduced staining for GM2 ganglioside in Fig. 6 and Fig. S6, this was inconsistently present in the other two pairs of dKO and KIKO mice that were studied. The similarities in ganglioside content were further confirmed by tandem MS/MS, in which both GM2 and GD2 were significantly elevated and there was no significant difference in the levels in the dKO and KIKO models (Fig. 5A and B).

Lyso-GM2 was found to be significantly elevated in dKO and KIKO mice compared to CON mice (Fig. 5A). In humans, higher plasma lyso-GM2 values are associated with more severe GM2 accumulation and therefore phenotype (Kodama et al., 2011). Mouse models of SD were found to have lyso-GM2 accumulation in the brain, which decreased on treatment with intracerebroventricular injection of modified Hex B enzyme (Welford et al., 2022). Lyso-gangliosides are suspected to contribute to immune reactivity and pathology like other lyso-compounds, although more work in this area is required to understand their specific role (van Eijk et al., 2020). Given these findings, it is important that a significant decrease in lyso-GM2 and increase in GM3 was found in the brains of KIKO mice when compared to the dKO mice (Fig. 5A). The decrease in lyso-GM2, increase in GM3 and *Hexa* transcripts all indicate that residual HexA appears to be made in the KIKO model even though this was not reflected in the neurological phenotype or enzyme assay results.

The *HEXA* mutation causing the Gly269Ser substitution in the α-subunit of HexA (Navon and Proia, 1989; Paw et al., 1989) is the most frequent cause of adult-onset GM2 gangliosidosis in both Jewish and non-Jewish populations (Navon et al., 1990; Neudorfer et al., 2005). Fibroblast-culture based studies demonstrated that the Gly269Ser substitution was the most sensitive to the chaperone pyrimethamine among several tested GM2-gangliosidosis mutations (Maegawa et al., 2007). Pyrimethamine was tested as a therapy in some late-onset Tay-Sachs patients but increases in HexA activity were not sustained (Osher et al., 2015). These findings suggested that the Gly269Ser substitution could be a candidate for the development of new chaperones, and this would be facilitated by the availability of a model that made *Hexa*-encoded protein. Another attractive therapy option for GM2 gangliosidoses is gene correction with CRISPR/Cas9, which has been successfully done in fibroblast and HEK 293T cell lines (Allende et al., 2018; Anzalone et al., 2019). However, no *in vivo* models exist that would be amenable to single base-pair gene correction using CRISPR/Cas9. In one study, fibroblasts taken from a SD patient containing both a splice site point mutation IVS10-2A>G on one allele and a 16 kb deletion on the other were treated using a corrected template for the IVS10-2A>G mutation and homology directed repair via CRISPR/Cas9 (Allende et al., 2018). Restoration of one allele increased β-hexosaminidase activity to 50% of WT, well above the disease threshold. Correction of single base pairs and small deletions have been reproduced in similar cell culture studies (Leal et al., 2022) but have not been tested in mouse models.

We examined the molecular and biochemical consequences of the Hexa Gly269Ser KI allele in mice to determine if the KIKO model synthesized *Hexa*-encoded protein. Despite the normal level of *Hexa* mRNA for the *Hexa* Gly269Ser KI, both the *Hexa* KI and KO mice exhibited similarly low levels of HexA activity (Fig. 1A). This low-level activity has been seen previously in *Hexa^−/−^* mice and resulted from HexB which has a high Km for 4-MUGS (Phaneuf et al., 1996). This result was not unexpected as even in humans with adult-onset forms of TSD, the level of activity was in the range of TSD homozygotes using the classical heat inactivation assay and mature α-subunit protein was undetectable (Navon et al., 1986). We were unable to find an antibody sufficiently sensitive to detect the α-subunit of HexA in mouse tissues.

The *Hexa* G269S substitution in the mouse resulted in a more severe form of disease compared to humans. It is likely that the G269S α-subunit protein is less stable in the mouse system. In humans, the Ser269 substitution in HexA's α-subunit appears to result in misfolding of the *α*-subunit, making it susceptible to destruction by the ER-associated degradation (ERAD) pathway. While some subunits escape ERAD, misfolding also appears to adversely affect the *α*-subunit active site, leading to between 4-8% residual activity in human cells (Lemieux et al., 2006). Non-conserved regions account for ∼20% of the mouse *Hexa*-encoded protein, which may create additional unfavourable interactions with G269S mutation, a greater degree of ERAD, or even further reductions to activity compared to humans. G269S may also result in an unstable subunit association in mouse HexA enzyme. This has been shown in certain human mutations that result in pseudodeficiency, in which some α-subunit proteins fold well enough to avoid ERAD and associate unstably with *β*-subunits, particularly under laboratory conditions (Cao et al., 1997). Interestingly, overexpression of mutant human G269S α-subunits with normal *β*-subunits in COS cells results in a heat labile HexA which is unstable at 37°C (Brown and Mahuran, 1993). Further destabilisation in mice could result in either diminished *α*-subunits due to misfolding and ERAD or produce a highly unstable enzyme with low activity in the lysosome and in laboratory enzyme testing.

*Neu3* KO is required for the accumulation of GM2 in the models described herein, however the species-specific differences in NEU3 activity limit the similarity of the dKO and KIKO model to human TSD. NEU3 is known to have many endogenous substrates, including a variety of ganglioside substrates, along with evidence of involvement in specific signal transduction pathways (Miyagi and Yamamoto, 2022). Indeed, in mouse models of GM1 gangliosidosis, a similar bypass mechanism exists in which NEU3 acts on GM1 to produce GA1 ganglioside. To produce a severe model resembling human GM1 gangliosidosis, Neu3 KO was also required, indicating its role in the degradation of other gangliosides in mice (Allende et al., 2023). Additionally, humans and mice with *HEXA/Hexa* mutations do not accumulate GM2 in their visceral organs, whereas previous dKO models with *Hexa* and *Neu3* mutations showed moderate visceral accumulation more consistent with SD models (*Hexb^−/−^*) (Phaneuf et al., 1996; Sango et al., 1995)*.* Since other substrates can be degraded by NEU3 *in vivo*, the *Hexa^+/−^Neu3^−/−^* controls are most suitable to compare to the dKO and KIKO models when interpreting the results of the ganglioside mass spectrometry. It is difficult to determine how much impact *Neu3* gene deletion alone has on phenotype in this multi-gene system, since *Neu3* KO does not impact survival or ganglioside accumulation in mice. However, its modulating role in the disease process in mice is clear. This modulating role may change HexA enzyme activity disease threshold compared to humans, and thus the phenotypic impact human mutations may have in the context of mouse biochemistry. Further investigations will be required to determine this.

Until now, *Hexb* KO (SD) mice have been used for most studies of GM2 gangliosidosis therapies. Models suitable for testing chaperones that stabilize HexA or CRISPR-Cas9 based approaches that are designed for specific mutations have not been available in the mouse. The new model, designated *Hexa*KI*Neu3*KO, or KIKO, provides the opportunity to explore additional therapeutic options. Although we did not study the disease pathology in mice that were homozygous for only the Gly269Ser KI mutation, they would be expected to have a phenotype similar to *Hexa^−/−^* mice (Miklyaeva et al., 2004) and should also be useful in the investigation of single-base-pair gene correction therapies using CRISPR/Cas9 for adult-onset Tay-Sachs disease or as a model for assessing therapeutics aimed at the ERAD pathway. Additionally, this model may be suitable for studying the impact of lyso-GM2 on disease pathology as well as the impact of immunological responses to therapies in the presence of residual enzyme activity.

## MATERIALS AND METHODS

### Mice

All studies were performed under a protocol approved by the University of Manitoba Animal Care Committee and in a facility holding a certificate of Good Animal Practice from the Canadian Council on Animal Care. dKO and KIKO mice were paired to age, sex matched CON (either *Hexa^+/−^ Neu3^−/−^* or *Hexa^+/−^ Neu3^+/−^*) for all behavioural testing. Paired animals were housed together, except where fighting between animals prevented this, with long capped water bottles and food on cage floors to facilitate access. Mice were monitored on a weekly basis starting at 7-10 weeks for changes in signs of physical, behavioural, and neurological function. Body weight and signs of tremor, fur ruffling, eye conditions or skin wounds were recorded every monitoring session, along with a 5 s tail suspension to assess hindlimb extension reflex. HEP was defined in accordance with animal care protocols, and mice were euthanised by isoflurane overdose within 24 h when one or more of the following conditions were met: (1) 15% weight loss from maximum weight, (2) excessive shaking or seizure activity, (3) isolating behaviour, (4) inability to access food or water due to reduced mobility, and (5) skin wounds resulting from fighting or self-mutilation deemed by veterinary staff to be untreatable.

### Generation of *Hexa* KO/KI and *Neu3* KO mice using CRISPR/Cas9

Guided by previous studies (Seyrantepe et al., 2018), we aimed to generate a TSD-like mouse model by combining HexA α-subunit (*Hexa*) and neuraminidase 3 (*Neu3*) deficiency. Further by creating a *Hexa* KI allele for the Gly269Ser mutation that could be combined with the *Neu3* KO allele, we hoped to generate a less severe phenotype and later-onset form of GM2 gangliosidosis.

To alter *Hexa* we used the Custom Alt-R^®^ CRISPR/Cas9 guide RNA design tool at https://www.idtdna.com/pages to analyze approximately 50 bp of sequence encompassing the Gly269 encoding sequence in exon 7 of *Hexa* (NC_000075.7:59,466,606–59,466, 740).

A guide adjacent to the sequence to be modified (5′ACTGCAGCTCCATTCTTACC-3′) was synthesized as an Alt-R^®^ CRISPR/Cas9 crRNA by Integrated DNA Technologies (IDT; Coralville, IA, USA). A single-stranded DNA (ssDNA) donor designed to introduce the NM_010421.6 c.805G>A P.G269S mutation 5′tgtgctggcagaatttgacactcctggccacactttgtc*a*tgggggccaAgtaagaatggagctgcagttggaggcgtctgctaaaagaggggctccaggg-3′ was also provided by IDT. The base that was altered to introduce the amino acid substitution and simultaneously destroy the CRISPR Pam site in the targeted DNA is capitalised. A second base change of c.795C>A (italics) does not alter the coding sequence but destroys a second ScrF1 site to facilitate PCR-based screening.

Changes were introduced into the mouse genome using the Alt-R^®^ CRISPR/Cas9 System from IDT and based on a previous method (Qin et al., 2015) but using *in vitro* fertilised C57BL/6N embryos as described previously (Hashimoto et al., 2016). Fertilised embryos were cultured for 2 h in potassium simple optimized medium (KSOM) and then groups of 20-40 fertilised embryos were electroporated in a 10 µl volume of Opti-MEM^®^ (Thermo Fisher Scientific) containing 50 ng/µl Cas9 (Alt-R^®^ S.p. Cas9 Nuclease V3), 200 ng/µl of the guide duplex prepared following the manufacturer's instructions, and 400 ng/µL of ss DNA donor). The electroporation was done using the BioRad Gene Pulser Xcell™ at 30 V, 1 s ON, 99 s OFF, for 12 cycles. Embryos were cultured to the 2-cell stage and transferred to CD1 pseudopregnant mice (0.5 dpc).

For screening of the offspring, a 217 bp amplicon including the region of *Hexa* that was edited was PCR amplified using forward primer 5′- CAACCCTGTCACTCACATCTAC -3′ and reverse primer 5′-CAAGAGCCAAGGCCAAGATA-3′. The amplicons were analysed on an 8% polyacrylamide gel with ScrF1 digestion to test for the desired c.805G>A mutation and without ScrF1 digestion to screen for heteroduplexes resulting from small insertions or deletions (Fig. S7A and B).

The same approach was used to generate a *Neu3* KO mouse. A CRISPR guide RNA targeting exon 2 of *Neu3* (NC_000073.7, 99,472,529–99,472,740), 5′-TGCGGGGCCATGTTACTGAG-3′ was identified and used to create insertions/deletions in embryos following the procedure above but without ss donor DNA. The region of *Neu3* targeted by the guide RNA was PCR-amplified using forward primer 5′-CTACTGATGGAGGCCACATTAC-3′ and reverse primer 5′-CTCCTCGGTCAAGTCTTTCAC-3′. The resulting 219 bp amplicons were screened by heteroduplex analysis as described for *Hexa* above (Fig. S7C,D).

Based on the PCR-screening of 24 *Hexa*-targeted and 18 *Neu3*-targeted mice, alleles of interest were sequenced. Six founding mice with *Hexa* KI and/or *Hexa* KO alleles and five mice with *Neu3* KO alleles were chosen for further breeding (Tables 1 and 2). These mouse lines are available upon request.

### Genotyping

DNA extracted from mouse ear clippings (Laird et al., 1991) was used for PCR amplification using the same primers and strategies specified above for screening *Hexa* and *Neu3*. Insertions or deletions were detected by heteroduplex analysis where PCR products were denatured and annealed with an equal quantity of WT PCR product to form heteroduplexes (Fig. S7E,F) and the c.805G>A mutation was detected using ScrFI digestion (Fig. S7,G). The PCR products, with and without annealing to WT DNA, were then separated by polyacrylamide gel electrophoresis. DNA heteroduplexes resulting from the mismatches between the mutant and WT sequence result in slower migrating bands (heteroduplexes) that are unique for each mutation (Fig. S7A,C,D,E,F).

### Tissue collection and processing

Adult mice (>P21) were euthanised by isoflurane inhalation (40% v/v in polyethylene glycol), and blood was collected by cardiac puncture. One hemisphere of the brain was flash frozen on dry ice while the other was fixed in 10% formalin for embedding. Frozen brain tissues were homogenized in phosphate buffered saline (PBS), pH 6.6 containing mammalian protease inhibitor cocktail (1:500, Sigma-Aldrich P8340) using a Sonic Dismembrator (Model 100, Thermo Fisher Scientific). Aliquots of tissue lysates were frozen at −80°C.

### GM2 analysis by thin layer chromatography

Gangliosides were isolated from frozen brain lysates using methanol/chloroform extraction and separated by thin layer chromatography as described previously (Walia et al., 2015), except that the partitioning was done using a hydrophobic column (Strata^®^ C18-E; 55 µm, 70 Å), and gangliosides were visualized with orcinol spray. The plate was photographed and GM2 and GM1 bands were analysed by densitometry using Image J software.

### Lipid isolation and ganglioside quantification with LC-ESI-MS/MS

Liquid chromatography coupled with electrospray ionization-tandem mass spectrometry (LC-ESI-MS/MS) was employed for ganglioside analysis and quantification. Lipid was extracted from 100 µg of protein equivalent of mouse brain homogenate (KIKO *n*=4, dKO *n*=4, CON; 1 WT *Hexa* allele *n*=5, 1 WT *Neu3* allele *n*=2, double Het *n*=1) as previously described (Saville et al., 2017) but with the addition of 20 pmol of N-omega Cd3-octadecanoyl monosialoganglioside (GM1 d3) and N-omega-CD3-octadecanoyl disialoganglioside (GD3 d3) (Matreya Inc, Pleasant Gap, PA, USA) as internal standards for the monosialo- and disialo- gangliosides, respectively. Gangliosides were measured by LC-ESI-MS/MS as described (Saville et al., 2017). Lipid nomenclature was as per LIPID MAPS (www.lipidmaps.org) with the sum composition of the sphingolipid base and fatty acyl chain reported for all gangliosides.

### Enzyme assays

Brain sonicates were divided into supernatant and pellet by centrifugation at 17,000× ***g*** for 10 min and protein concentrations were determined by the Bradford assay. HexA activity in supernatants was determined using 1 mM 4-MUGS (Toronto Research Chemicals) as described (Sharma et al., 2001). Reactions were quenched with 20 volumes of GC buffer (pH 10.2), and fluorescence units were measured using a SpectraMax M2 plate reader (excitation: 365 nm; emission: 450 nm) using methylumbelliferone in GC buffer to generate a standard curve. Sialidase was determined from the pellet resuspended in PBS. Aliquots (150, 225 and 300 µg of protein) were incubated in sodium acetate buffer (pH 4.4, 0.2 M) with 0-200 µM of the sialidase inhibitor Zanamivir (ZANA, 4-Guanidino-2,4-dideoxy-2,3-dehydro-N-acetylneuraminic acid, Sigma-Aldrich) for 15 min (Guo et al., 2018). Samples were then assayed with 200 µM 4MU-NANA (4-methylumbelliferyl N-acetyl-a-D-neuraminic acid, sodium salt Sigma-Aldrich) in 1% dimethylformamide at 37°C for 1 h. Reactions were quenched with 15 volumes of GC buffer (pH 10.2), and fluorescence was measured as described above.

### *Hexa* transcript analysis

Total RNA was extracted using the illustra^TM^ RNAspin Mini Isolation kit (GE, cat # 25-0500-87) from 30 mg of liver tissue from WT (*Hexa^wt/wt^*, *n*=2), HET (*Hexa^em1.2 /wt^*, *n*=2), KI (*Hexa^em2.2^*^/*em2.2*^, *n*=3) and KO (*Hexa^em1.2/ em1.2^*, *n*=3) mice, cDNA was produced using 1 µg of RNA with Luna Script RT master mix (New England Biolabs) containing oligo-dt and random hexamers and repeated in parallel using the Luna Script no RT control. The *Hexa* cDNA product was then PCR amplified along with genomic DNA (*Hexa^wt/wt^*, *n*=1) using forward primer 5′- CAACCCTGTCACTCACATCTAC -3′ targeting exon 7 and reverse primer 5′ -CCCTCCCAGGTGGAGATAAA -3′ targeting exon 8. PCR fragments were separated on an 8% polyacrylamide gel.

### Behavioural and motor analysis

For hindlimb extension reflex tests, mice were suspended by the tail for 5 s and hindlimb extension reflex was scored according to Table S1. Open field and gait analysis was performed at approximately 10, 14, 18 and 22 weeks of age. Mice were acclimated for at least 30 min to the testing room and then placed in a 50 cm^2^ square open field arena with white floor and recorded for 10 min using a webcam and Bandicam video recording software (version 4.6.5.1757). ANYMaze software (version 6.33, Stoelting Co., Wood Dale, IL, USA) was used for video tracking and further analysis. Mice were rested for at least 30 min in their home cage before gait analysis was performed. Non-toxic paint was applied to all feet before the mice were released into a paper lined 10 cm wide passage with a dark chamber at the end to encourage forward movement. Five or more consecutive strides with normal walking were acquired for each mouse. Stride, stance, and sway distances were measured three times and averaged.

### Immunohistochemistry

Immunohistochemistry was performed on 5 µm sagittal brain sections after deparaffinization followed by antigen retrieval in 10 mM sodium citrate (pH 6.0) at 95°C for 20 min. After pre-treatment with 3% H_2_O_2_ for 10 min, and 3% bovine serum albumin (BSA) in Tris-buffered saline (TBS) for 1 h, sections were treated with the Avidin/Biotin Blocking Kit according to the supplier (Vector; VECTSP2001). Sections were incubated overnight at 4°C with mouse monoclonal anti-GM2 IgM (1:2000, Tokyo Chemical Industry America Clone Number MKI-16, A2576, Lot #3HHPF) diluted in blocking solution or in blocking solution only for negative controls (CON). After washing with TBS, the slides were incubated with biotin-conjugated polyclonal goat anti-mouse IgM (1:2000, Jackson Labs, 115-065-020). The slides were then washed and treated with the ABC complex solution according to the supplier (Vector; PK4000), rinsed briefly in TBS, and then incubated for 2 min with ImmPACT DAB EqV Peroxidase Substrate (Vector Labs, SK-4103). Slides were washed in distilled water (5 min) and counterstained with 0.5% methyl green in 0.1 M sodium acetate buffer for 5 min. This was followed by dehydration and mounting using Permount (Fisher Scientific). Images were taken using Zeiss Axioimager at 20× and 63×.

### Statistical methods

Data were analysed using GraphPad Prism version 10.4.1 (Dotmatics, Boston, MA, USA). The selected method of analysis is indicated for each figure. The sample sizes of three female and three male mice per group was estimated for our animal care committee to provide an 80% chance of finding a difference in the combined group compared to control mice if 70% of the animals reached a HEP within one year.

## Supplementary Material

10.1242/biolopen.062045_sup1 Supplementary information
