# Supplementary material for: Generation of mice with combined Hexa Gly269Ser KI or KO and Neu3 KO alleles to create new models of GM2 gangliosidoses
Source: Biol Open. 2025 Sep 30;14(9):bio062045. doi: 10.1242/bio.062045 (PMC12519549; doi:10.1242/bio.062045)
Supplement: Supplementary information [file biolopen-14-062045-s1.pdf]

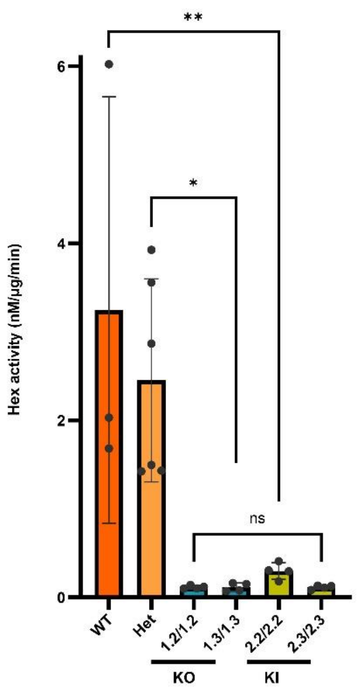

Fig. S1. HexA activity in CON, dKO and KIKO mice separated by genotype.

Brain lysates were assayed using the synthetic substrate 4-MUGS. Columns represent the mean ± s.d. activity. The alleles comprising the genotypes of the dKO and KIKO mice are shown. HexA activity is significantly reduced in the brains of dKO (n=4 per genotype) and KIKO (n=4 per genotype) mice compared to either WT (n=3) or HET (n=6) controls ($p < 0.006$, ** for WT and $p<0.03$, * for HET). However, there were no significant differences between dKO and KIKO models of any genotype. ns – non-significant. Statistics were generated using a one-way ANOVA with Sidak's multiple comparison testing.

Biology Open • Supplementary information

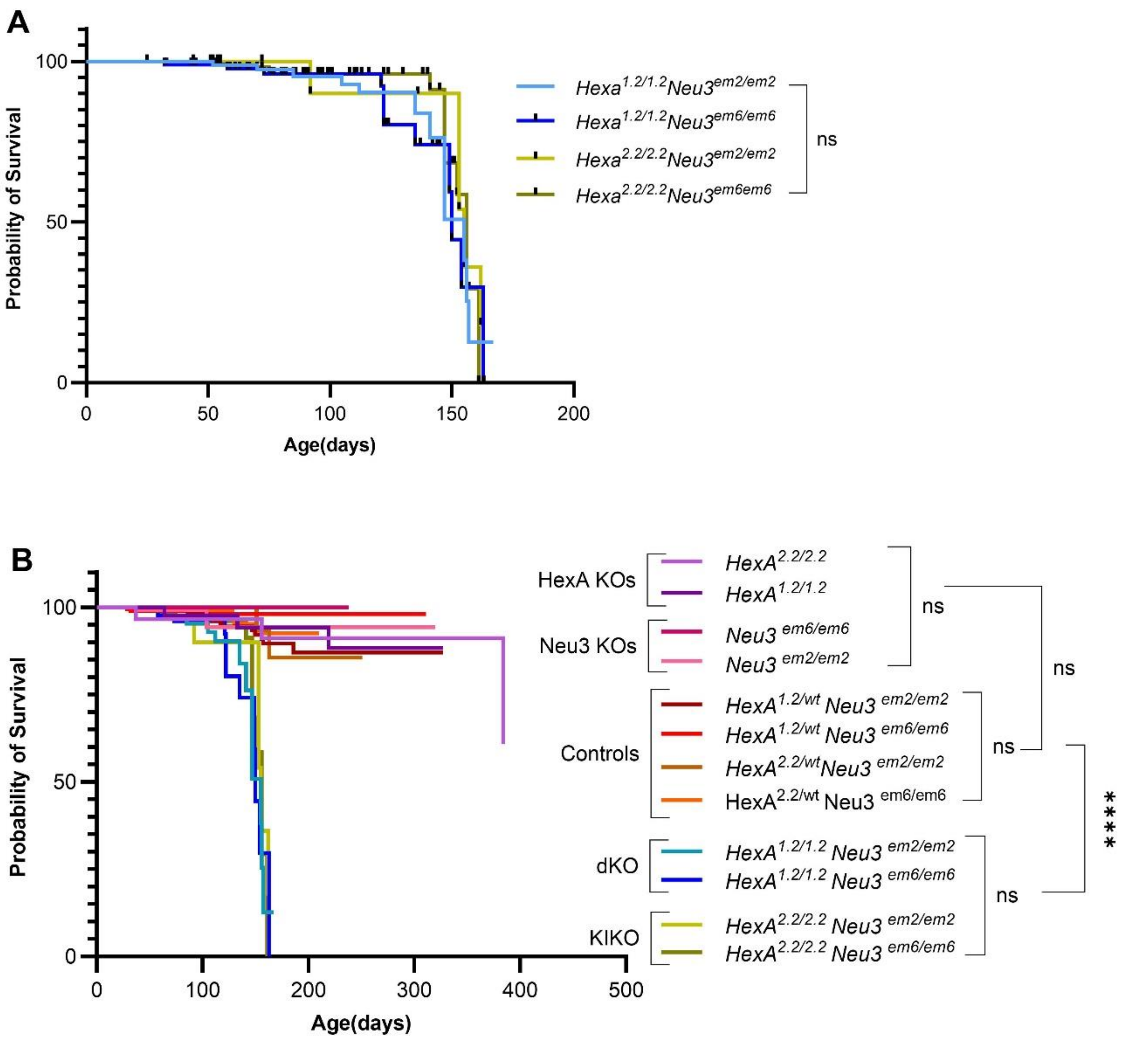

Fig. S2. Survival effects of different KO and KI alleles.

A. Kaplan-Meier survival analysis of dKO and KIKO mice with the *Neu3*^{em2} and *Neu3*^{em6} alleles. All dKO or KIKO mice homozygous for either the *Neu3*^{em2} (n=100 dKO and 12 KIKO) or *Neu3*^{em6} (n=101 dKO and 64 KIKO) allele were compared. Statistics were conducted using log-rank tests for individual group comparsions and p-values adjusted for multiple comparisons using Holm-Sidak method (α=0.05). The two *Neu3* alleles had no impact on survival probability. ns (non-significant). **B. Kaplan-Meier survival analysis of *Neu3* KO, dKO, KIKO and CON mice with the *Neu3*^{em2} and *Neu3*^{em6} alleles.** No differences were observed in the survival of mice with *Neu3*^{em2} (n= 131 KO, 100 dKO, 12 KIKO, 179 HET KO, 41 HET KI or *Neu3*^{em6} (n= 116 KO, 101 dKO, 64 KIKO, 186 HET KO, 132 HET KI) alleles. As expected, the only significant difference was between dKO or KIKO and CON mice ($p < 0.00001$, ****). Statistics were conducted as described in **A**.

Biology Open • Supplementary information

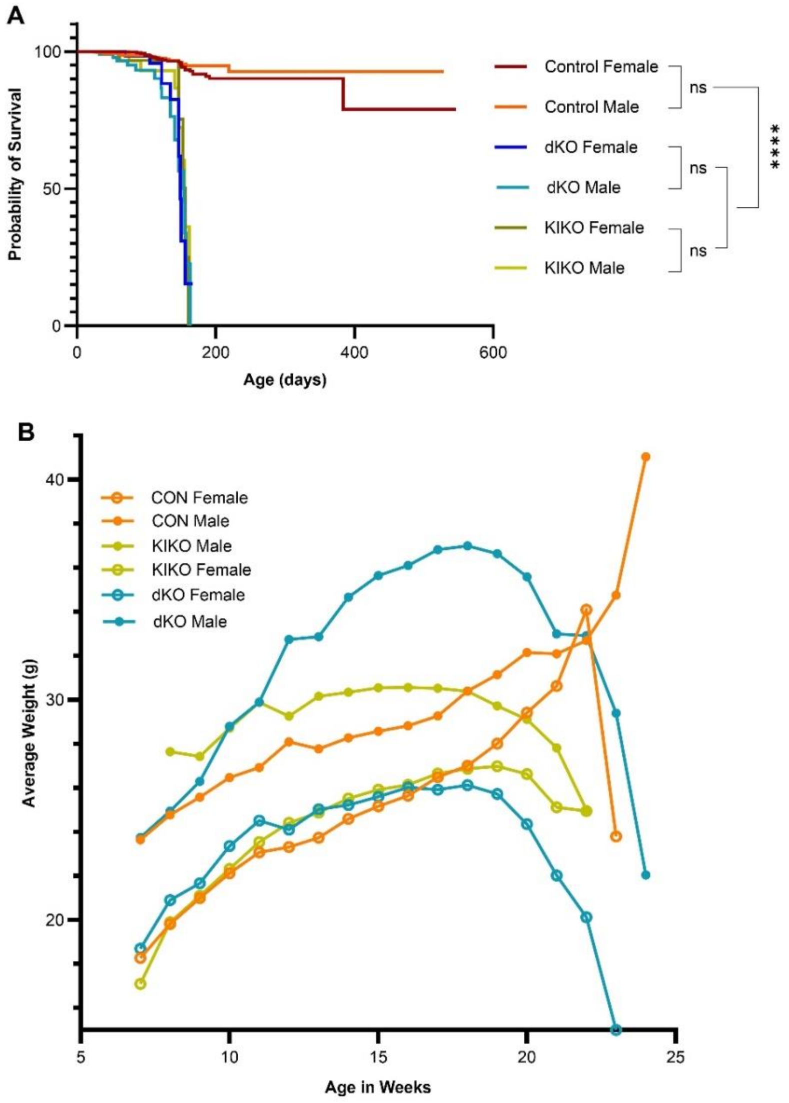

Fig. S3. Weight and survival analysis of male and female *HexaNeu3* dKO and *HexaNeu3* KIKO mice. A. Kaplan-Meier survival analysis. No difference in survival probability between male (n= 688 CON, 103 dKO, 36 KIKO) and female (n= 632 CON, 98 dKO, 40 KIKO) mice of any genotype was observed. Statistics were conducted using log-rank tests for individual group comparsions and p-values adjusted for multiple comparisons using Holm-Sidak method (α=0.05). **B. Weight trajectory of female and male dKO, KIKO and CON mice.** The weight of the mice was measured weekly starting between 7 and 10 wks of age until the HEP was reached. Lines connect the average weight for the male (n = 2-11 CON, 2-6 dKO, 2-5 KIKO) or female (n = 4-9 CON, 2-5 dKO, 2-4 KIKO) mice with each genotype. The number of animals at each time point may differ depending on when they were entering the study or being euthanized, with each group having their largest n values from 10 to 20 weeks.

Biology Open • Supplementary information

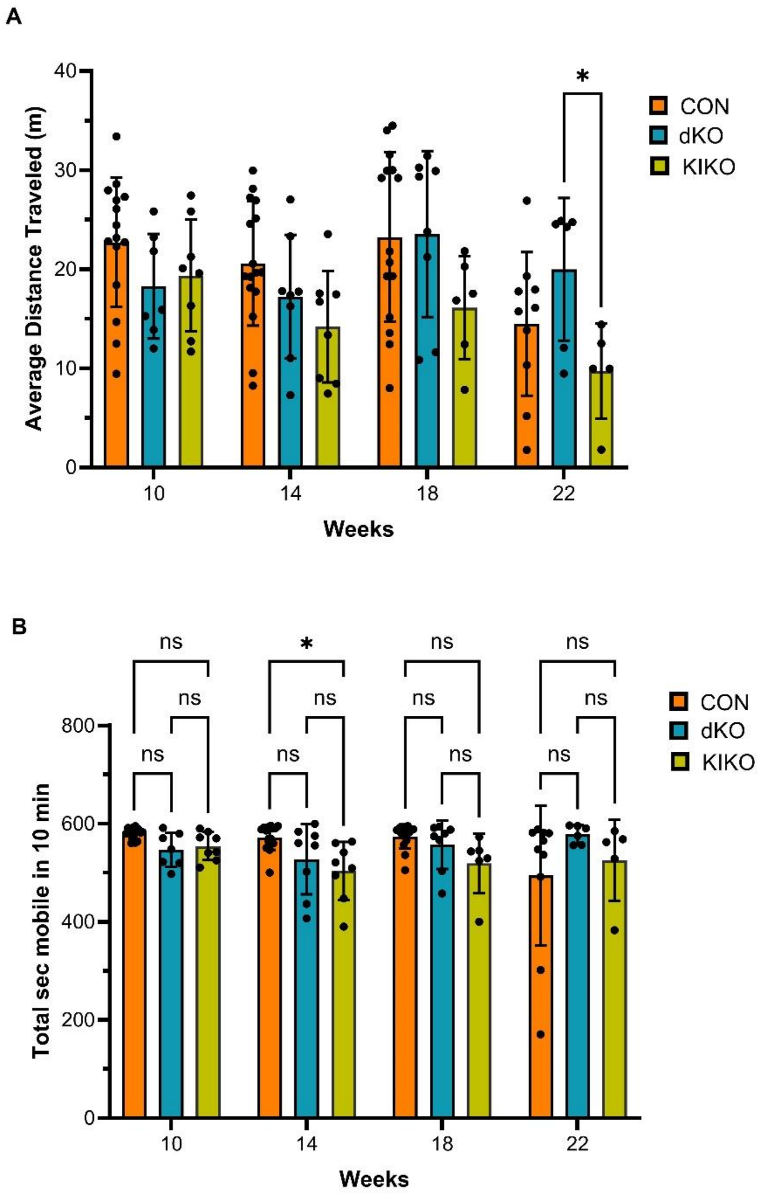

Fig. S4. Mobility Measures in dKO, KIKO and CON mice. A. Distance travelled. Distance travelled in the chamber was measured in meters during a 10 min test. Each column represents the mean ± s.d. of distance travelled. The distance travelled of dKO (n =6-8) and KIKO (n=5-8) mice was not significantly different when compared to CON (n=10-16) mice except for the 22 wk point ($p < 0.05$, *). ns (non-significant). Statistics were conducted using a mixed-effects model (REML) using Geisser-Greenhouse correction, with Tukey's multiple comparisons test for each comparison. **B. Time mobile.** Time mobile in the chamber was measured in seconds during a 10 min test. Animal numbers and statistics were described in **A**.

Biology Open • Supplementary information

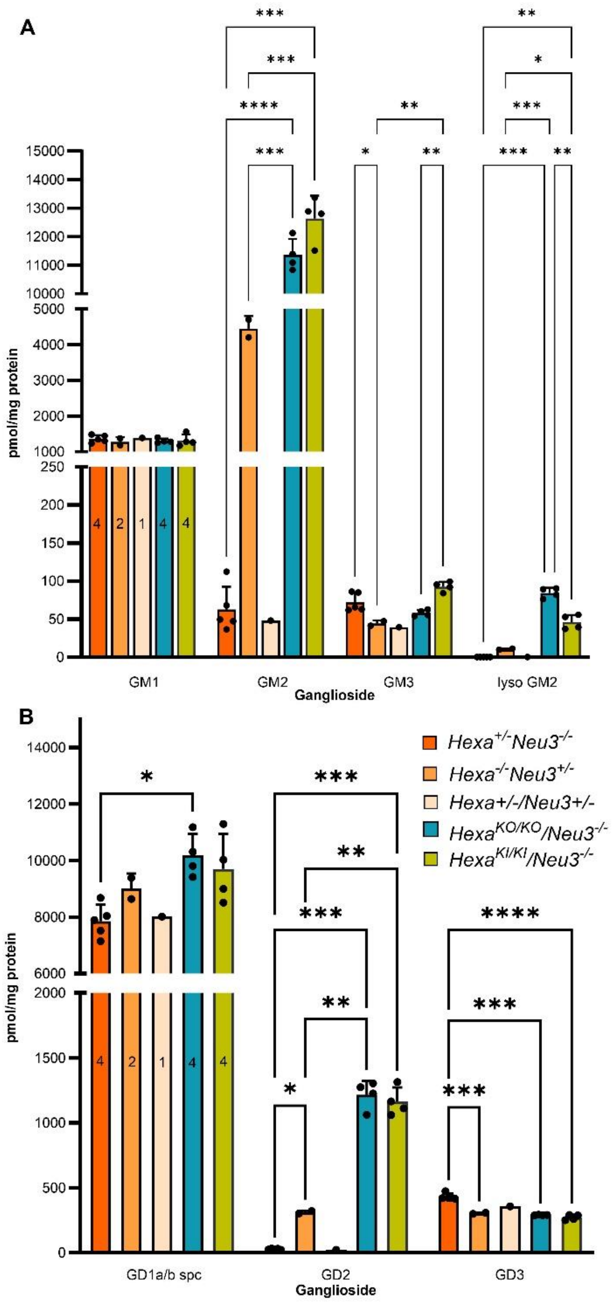

Fig. S5. Gangliosides detected in brain samples with controls separated by genotype. Brain gangliosides were identified by mass spectroscopy. Columns represent the average ganglioside level associated with each genotype shown in the legend. The sample n varies for each genotype and is indicated within the GM1 and GD1a/b columns. For statistical analysis the single *Hexa*$^{+/-}$ *Neu3*$^{+/-}$ sample was removed. Statistics were conducted using a two-way ANOVA with the Geisser-Greenhouse correction, with Tukey's multiple comparisions test for each comparision..* (p<0.05), ** (p<0.01), *** (p<0.001), **** (p<0.0001), ns (non-significant). **A. Monosialo gangliosides.** Monosialo-gangliosides with different lengths of fatty acyl chains were grouped together. **B. Disialo gangliosides.** Disialo-gangliosides also included different lengths of fatty acyl chains. GD1a/b spc (species) refers to the fact that several different ganglioside groups are included in this total.

Biology Open • Supplementary information

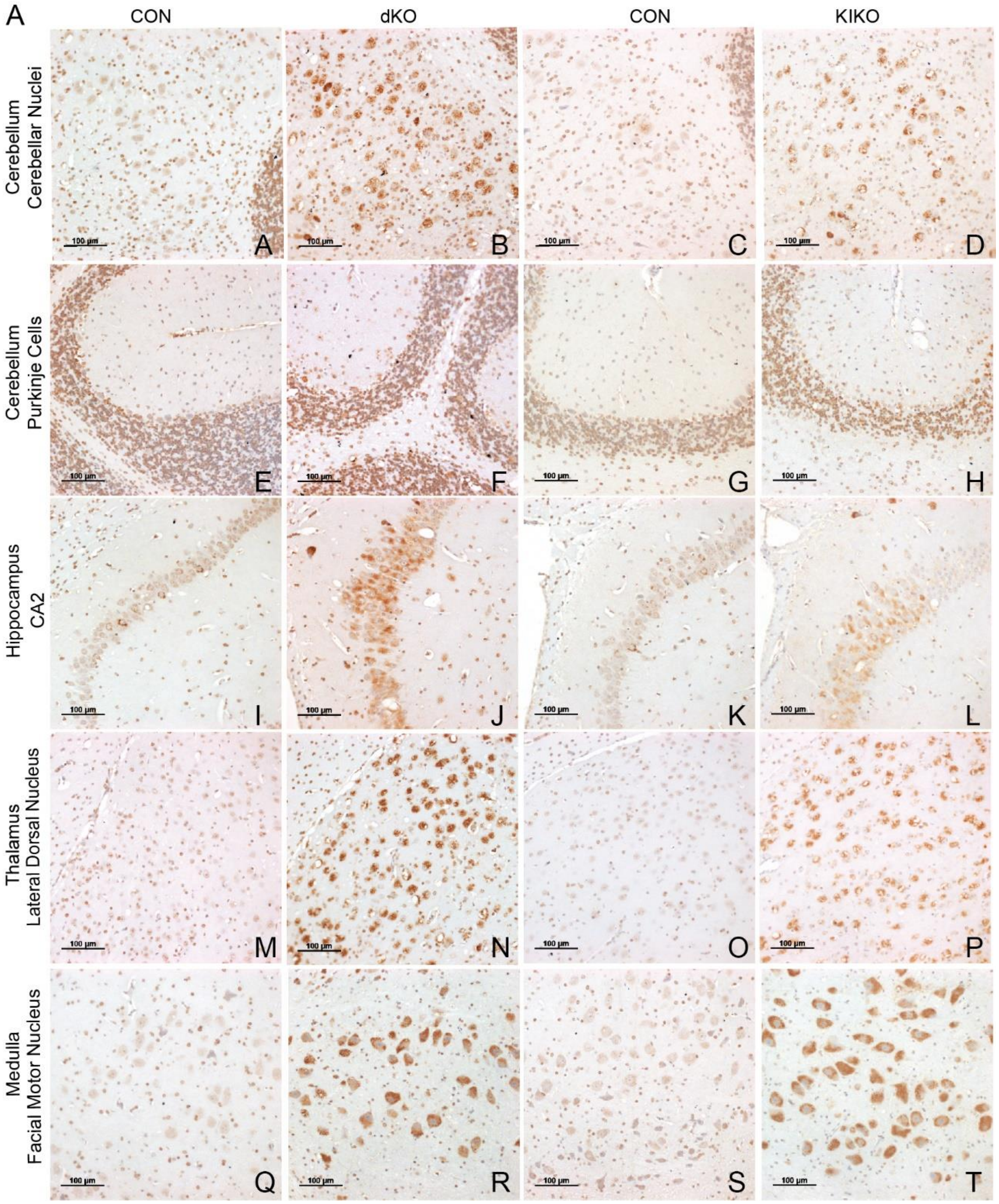

Biology Open • Supplementary information

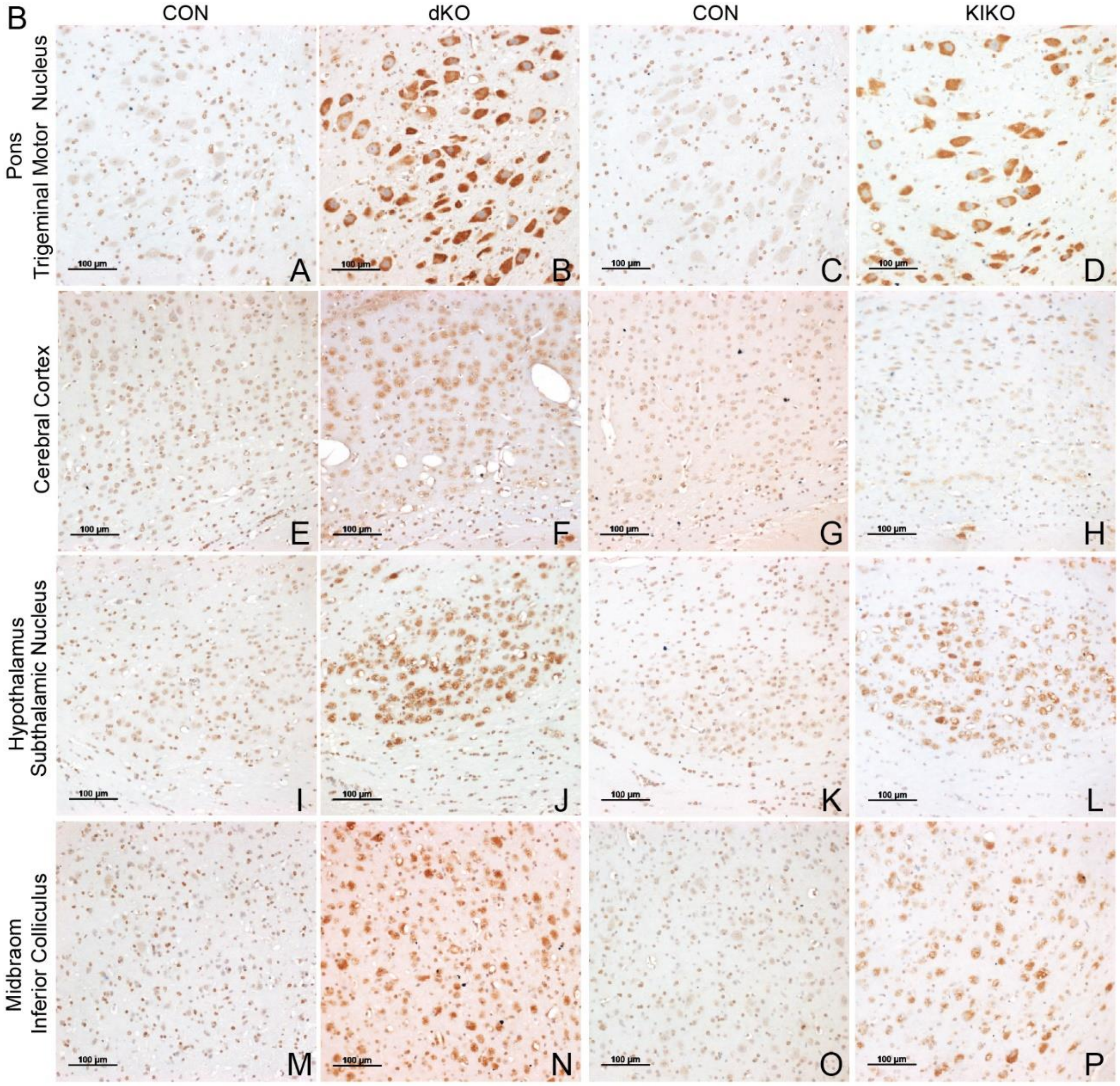

Biology Open • Supplementary information

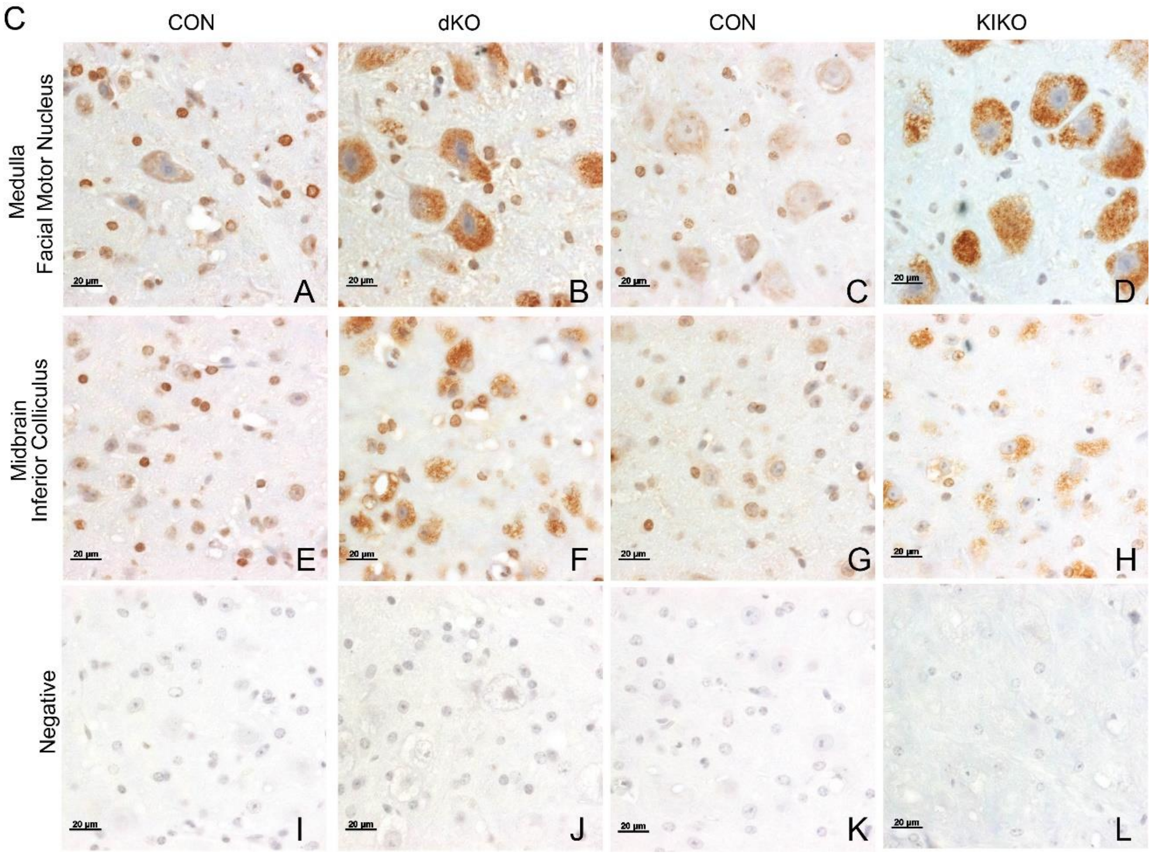

Fig. S6. Immunohistochemical detection of GM2 in CON, dKO, and KIKO brains. A and B. Low magnification (20X) images. Several regions of the brain from non-affected (CON), dKO and KIKO mice are shown. The red-brown signal indicates the presence of GM2 ganglioside. Scale bars are 100 µM. These images are representative of those of three different pairs of animals. For Fig. S6A, 63X magnification images taken from the same field of view of panel A, B, C, I, J, N, O, R and S are also shown in Fig. 6, panels E, F, G, I, J, N, O and Fig. S6C, panel B and C respectively. For Fig. S6B, 63X magnification images taken from the same field of view as Fig. S6B panel O can be found in Fig. S6C panel G. **C. High magnification (63X) images.** Several regions of the brain from non-affected (CON), dKO and KIKO mice are shown. Many images are taken within the same field of view as the 20X images in panels A and B. Data from Fig. S6C, panels B and C are also shown in Fig. S6A, R and S and data in Fig. S6C panel G are also shown in Fig. S6B, panel O. Red-brown signal indicates the presence of GM2 ganglioside. The negative control image shown at the bottom was treated identically to other samples but did not include the primary antibody. Scale bars are 20 µM. These images are representative of those of three different pairs of animals.

Biology Open • Supplementary information

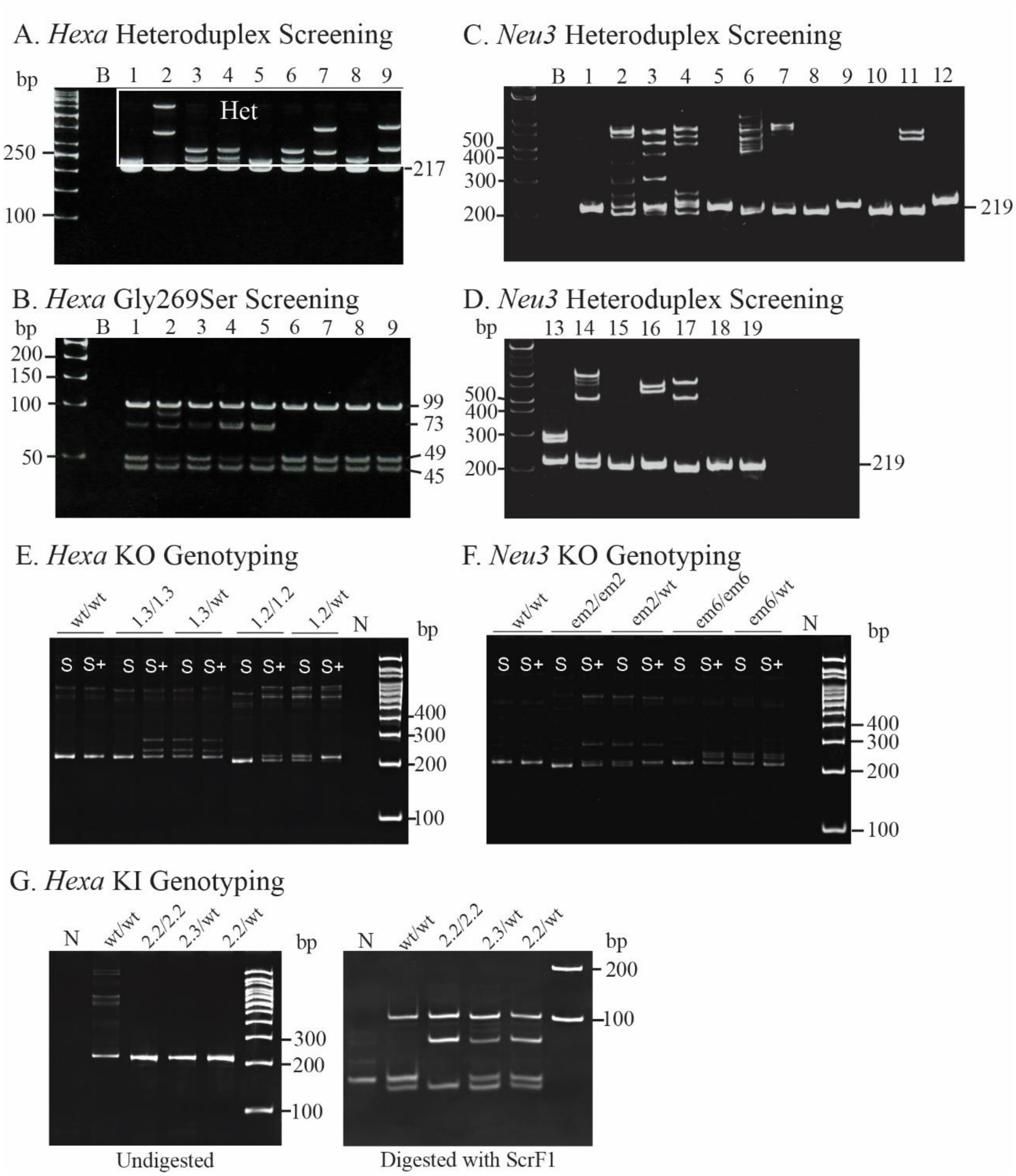

Fig. S7. Screening for and genotyping of *Hexa* and *Neu3* mutations. A. *Hexa* heteroduplex screening. To identify founders with mutant *Hexa* alleles, the region targeted with CRISPR/Cas9 was PCR-amplified and the PCR products were separated on an 8% polyacrylamide gel. A white rectangle surrounds the heteroduplexes in Founders 1 to 9 that indicate insertions/deletions greater than 2 bp are present on one allele of the sample. B- water blank. **B. *Hexa* Gly269Ser screening.** To identify founders with the Gly269Ser mutation, the targeted region of *Hexa* was PCR-amplified and digested with the ScrF1 enzyme whose recognition site is destroyed by the Gly269Ser-causing change. The c.805G>A mutation destroys an ScrF1site, resulting in

Biology Open • Supplementary information

99,49,46,16, and 8 bp fragments for the wild type sequence and 99, 75, and 46 bp fragments for the mutant Gly269Ser-encoding sequence. The presence of the 73 bp band in samples 1-5 indicates the G269S substitution is present. Samples 2, 4, and 5 appear to be homozygous for this change although sample 2 also appears to have an additional mutation. **C and D. *Neu3* heteroduplex screening.** To identify Founders with mutant Neu3 alleles, the region targeted with CRISPR/Cas9 was PCR-amplified and the PCR products were separated on an 8% polyacrylamide gel. PCR products with increased or decreased size compared to the expected 219 bp, or the presence of heteroduplexes indicated mutations for further follow up. **E and F. *Hexa* and *Neu3* KO genotyping.** Polyacrylamide gel electrophoresis of *Hexa* or *Neu3* KO PCR products were analyzed for the presence of mutant alleles using heteroduplex analysis where the experimental PCR products are annealed with wild type DNA prior to separation. PCR products that are annealed (S+) or not annealed (S) with product are examined. Wildtype mice do not display heteroduplexes in either S or S^+, while heterozygous mice display heteroduplexes in both. KO mice display heteroduplexes in S^+ only. **G. *Hexa* KI Genotyping.** PCR products without (left) and with (right) ScrFI digestion were separated on an 8% polyacrylamide gel as in (B). For ScrFI digest of *Hexa* KI PCR products, appearance of a 73 bp band indicates the G269S mutation. wt/wt (99bp, 49bp, 45bp), 2.2./2.2 (99bp, 73bp, 45bp), 2.3/wt and 2.2/wt (99bp, 73bp, 49bp, 45bp). L: 100bp Ladder (ThermoFisher); N: Negative Control, S: PCR product of sample DNA, S^+: PCR product of sample DNA + WT DNA PCR product.

Biology Open • Supplementary information

Table S1. Scoring of hindlimb flexion response on tail lift, adapted from Miedel et al (2017).

Score	Description
0	No limb clasping; normal extension response.
1	One hindlimb exhibits incomplete extension and intermittent loss of mobility; toes exhibit a normal splay.
2	Both hindlimbs exhibit incomplete extension and intermittent loss of mobility; toes exhibit a normal splay
3	Both hindlimbs exhibit intermittent clasping/flexion, with curled toes and intermittent loss of mobility.
4	Both hindlimbs exhibit clasping/flexion, or are crossed, with curled toes and persistent immobility.

Biology Open • Supplementary information

Table S2. Total ganglioside quantification in pmol/mg protein.

	Genotype					
	Hexa$^{em2.2/em2.2}$ *Neu3*$^{em6/em6}$	*Hexa*$^{em1.2/em1.2}$ *Neu3*$^{em2/em2}$	*Hexa*$^{em1.2/+}$ *Neu3*$^{em2/em2}$	*Hexa*$^{em2.2/em2.2}$ *Neu3*$^{em6/+}$	*Hexa*$^{em2.2/+}$ *Neu3*$^{em6/+}$	*Hexa*$^{em2.2/+}$ *Neu3*$^{em6/em6}$
N-Values	4	4	4	2	1	1
Ganglioside						
GD1a/b 36:1	7674	7646	5604	5904	5238	4663
GD1a/b 38:1	1827	2275	2114	2827	2522	2610
GD1a/b 40:1	134	166	158	206	178	175
GD1a/b 42:2	52	69	50	60	54	55
GD1a/b 34:1	21	23	15	19	20	15
Total GD1a/b	9707	10179	7941	9015	8012	7519
GD2 36:1	796	791	18	186	15	17
GD2 38:1	338	391	10	115	8	10
GD2 40:1	24	28	n.d.	8	n.d.	n.d.
GD2 42:2	5	7	n.d.	3	n.d.	n.d.
GD2 total	1163	1217	28	312	23	27
GD3 34:1	3	2	3	2	2	2
GD3 36:1	187	193	271	188	226	281
GD3 38:1	44	47	107	62	88	140
GD3 40:1	16	21	20	24	19	23
GD3 42:2	23	28	20	28	22	26
GD3 total	272	290	421	304	358	473
GM1 34:1	41	47	33	36	30	32
GM1 36:1	1008	999	1074	988	1085	1025
GM1 38:1	219	206	232	223	241	257
GM1 40:1	27	28	16	21	17	20
GM1 42:1	7	7	3	2	4	2
GM1 42:2	15	17	9	9	9	9
GM1 Totals	1317	1304	1369	1279	1386	1344
GM2 34:1	328	366	2	71	1	1
GM2 36:1	8252	7314	49	3060	34	36
GM2 38:1	3688	3301	16	1222	13	11
GM2 40:1	283	287	n.d.	76	n.d.	n.d.
GM2 42:1	33	32	n.d.	9	n.d.	n.d.
GM2 42:2	58	62	n.d.	14	n.d.	n.d.
GM2 totals	12642	11362	66	453	48	47
GM3 34:1	3	2	3	1	1	1
GM3 36:1	51	30	45	23	24	43
GM3 38:1	26	16	14	9	6	14
GM3 40:1	8	5	6	6	6	3
GM3 42:2	5	5	6	5	3	5
GM3 total	93	58	74	44	39	65
Lyso GM2	46	84	n.d.	10	n.d.	n.d.

n.d.- not detected
\+ - wild type

Biology Open • Supplementary information

REFERENCES

Miedel, C. J., Patton, J. M., Miedel, A. N., Miedel, E. S. & Levenson, J. M. Assessment of spontaneous alternation, novel object recognition and limb clasping in transgenic mouse models of amyloid-β and tau neuropathology. *Journal of Visualized Experiments* **2017**, 1–8 (2017).
